# Supplementary material for: A CRISPR-based ultrasensitive assay detects attomolar concentrations of SARS-CoV-2 antibodies in clinical samples
Source: Nat Commun. 2022 Aug 9;13:4667. doi: 10.1038/s41467-022-32371-4 (PMC9361972; doi:10.1038/s41467-022-32371-4)
Supplement: Supplementary file 1 — Supplementary Information [file 41467_2022_32371_MOESM1_ESM.pdf]

Supplementary Information

A CRISPR-based ultrasensitive assay detects attomolar concentrations of SARS-CoV-2 antibodies in clinical samples

Yanan Tang,[1,#] Turun Song,[2,#] Lu Gao,[1] Saifu Yin,[2] Min Ma,[2] Yun Tan,[1] Lijuan Wu,[3] Yang Yang,[4] Yanqun Wang,[5] Tao Lin,[2,*] and Feng Li[1,4,*]

Table of Contents

S1. UCAD probe design and optimization…………………………………………………........ S4

Supplementary Table 1. DNA sequences and modifications………………..............…………….……... S4

Supplementary Figure 1 | Optimization of probe design………………………….……………………..…. S5

Supplementary Figure 2 | Optimization of T4 polymerase…………….……………...……….…….…. S6

Supplementary Figure 3 | Optimization of UCAD probe concentration……….…………....……….….. S7

Supplementary Figure 4 | Sensitivity of UCAD in buffer……………...…………….……...……………. S8

Supplementary Figure 5 | PAGE analysis of UCAD……..………………………………………………. S9

Supplementary Figure 6 | Sensitivity of UCAD in human serum samples………………….……….. S10

Supplementary Figure 7 | Specificity of UCAD……………………………………………….…..….. S11

Supplementary Figure 8 | Validation of SARS-CoV serum samples……………………….….………. S12

Supplementary Figure 9 | Modularity of UCAD………..……………………………………………… S13

S2. Demographic information for clinical validation of UCAD...................................... S14

Supplementary Table 2. Demographic characteristics of 85 KTRs…………………………….……….. S14

S3. UCAD and cellularanalysis of KTRs……………………………………...……….. S15

Supplementary Figure 10 | Correlation of anti-RBD level with gender and age………..……………..… S15

Supplementary Figure 11 | Flow Cytometric analysis of KTRs…...……....…………………….………. S17

Supplementary Figure 12 | Anti-RBD positive v.s. negative KTRs……...……..……………..………. S19

Supplementary Figure 13 | Anti-RBD positive KTRs v.s. anti-RBD positive health participates...…. S21

Supplementary Figure 14 | Flow cytometry analysis of B cell and Th cell subsets in patient sample P1.. S23

Supplementary Figure 15 | Flow cytometry analysis of B cell and Th cell subsets in patient sample P2.. S24

Supplementary Figure 16 | Flow cytometry analysis of B cell and Th cell subsets in patient sample P3.. S25

Supplementary Figure 17 | Flow cytometry analysis of B cell and Th cell subsets in patient sample P4.. S26

Supplementary Figure 18 | Flow cytometry analysis of B cell and Th cell subsets in patient sample P5.. S27

Supplementary Figure 19 | Flow cytometry analysis of B cell and Th cell subsets in patient sample P6.. S28

Supplementary Figure 20 | Flow cytometry analysis of B cell and Th cell subsets in patient sample P7.. S29

Supplementary Figure 21 | Flow cytometry analysis of B cell and Th cell subsets in patient sample N1.. S30

Supplementary Figure 22 | Flow cytometry analysis of B cell and Th cell subsets in patient sample N2.. S31

Supplementary Figure 23 | Flow cytometry analysis of B cell and Th cell subsets in patient sample N3.. S32

Supplementary Figure 24 | Flow cytometry analysis of B cell and Th cell subsets in patient sample N4.. S33

Supplementary Figure 25 | Flow cytometry analysis of B cell and Th cell subsets in patient sample N5.. S34

Supplementary Figure 26 | Flow cytometry analysis of B cell and Th cell subsets in patient sample H1.. S35

Supplementary Figure 27 | Flow cytometry analysis of B cell and Th cell subsets in patient sample H2.. S36

Supplementary Figure 28 | Flow cytometry analysis of B cell and Th cell subsets in patient sample H3.. S37

Supplementary Figure 29 | Flow cytometry analysis of B cell and Th cell subsets in patient sample H4………………………………………………………………………………………………… S38

Supplementary Figure 30 | Flow cytometry analysis of B cell and Th cell subsets in patient sample H5………………………………………………………………………………………………… S39

Supplementary Figure 31 | The B cell subsets distribution in lymphocytes of seven IgG$^+$/IgM$^+$ KTRs (P1-P7) and five IgG$^-$/IgM$^-$ KTRs (N1-N5)………………...…………………….……………………. S40

Supplementary Figure 32 | The Th cell subsets distribution in CD4$^+$ cells of seven IgG$^+$/IgM$^+$ KTRs (P1-P7) and five IgG$^-$/IgM$^-$ KTRs (N1-N5)……………………...………….……….……….……….…… S41

Supplementary Table 3. The description and phenotypes of investigated B cell subtypes and the medians with minimum and maximum of proportional in two KTR cohorts…………………...….... S42

Supplementary Figure 33 | UCAD analysis of KTRs with the 3rd dose of vaccine…........………..….. S43

S1. UCAD probe design and optimization

Supplementary Table 1. DNA sequences and modifications

Name	Sequence (5'→3')
**UCAD**	
TS Probe	TTC CTC ACC ATG TCT GAG GTA CTC CTT AAA GGT CGA GCT GGA C
NTS Probe	TTG TTG AGG TAA CCA ACT ATT TGT TAC TGT TGC TTG TGG CCG TTT ACG TCG CCG TCC AG
RPA F-primer	TTG TTG AGG TAA CCA ACT ATT TGT TAC TGT T
RPA R-primer	TTC CTC ACC ATG TCT GAG GTA CTC CTT A
dsDNA barcode (sense strand)	TTC CTC ACC ATG TCT GAG GTA CTC CTT AAA GGT CGA GCT GGA CGG CGA CGT AAA CGG CCA CAA GCA ACA GTA ACA AAT AGT TGG TTA CCT CAA CAA
dsDNA barcode (anti-sense strand)	TTG TTG AGG TAA CCA ACT ATT TGT TAC TGT TGC TTG TGG CCG TTT ACG TCG CCG TCC AGC TCG ACC TTT AAG GAG TAC CTC AGA CAT GGT GAG GAA
**CRISPR-Cas12a**	
crRNA	UUA UUU CUA CUC UUG UAG AUC GUC GCC GUC CAG CUC GAC C
Reporter for fluorescence readout	FAM-TTATT-BHQ-1
Reporter for lateral flow readout	FAM-TTATT-Digoxin

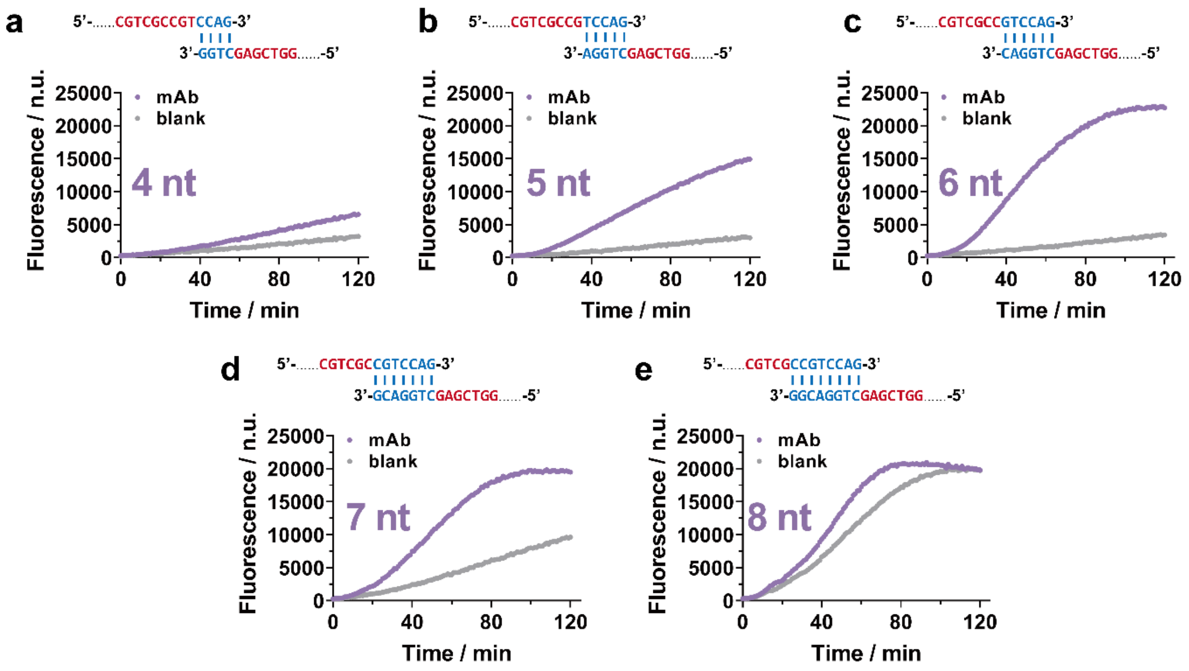

Supplementary Figure 1 | Optimization of probe design. Optimization of the length of the complementary domain of the TS and NTS probes from 4 nt to 8 nt in detecting anti-RBD mAb by UCAD. Because short complementary domains of the TS and NTS probes (4 nt and 5 nt) produce low fluorescence signal due to insufficient hybridization and probes with long complementary domains (7 nt and 8 nt) produce high fluorescence background due to target-independent hybridization, an optimal length of the complementary domain was found to be 6 nt. [mAb]= 100 fM, [TS probe] = 10 pM, [NTS probe] = 10 pM, [dNTPs]= 40 µM, [Cas12a] = 40 nM, [crRNA] = 40 nM, [Reporter] = 40 nM.

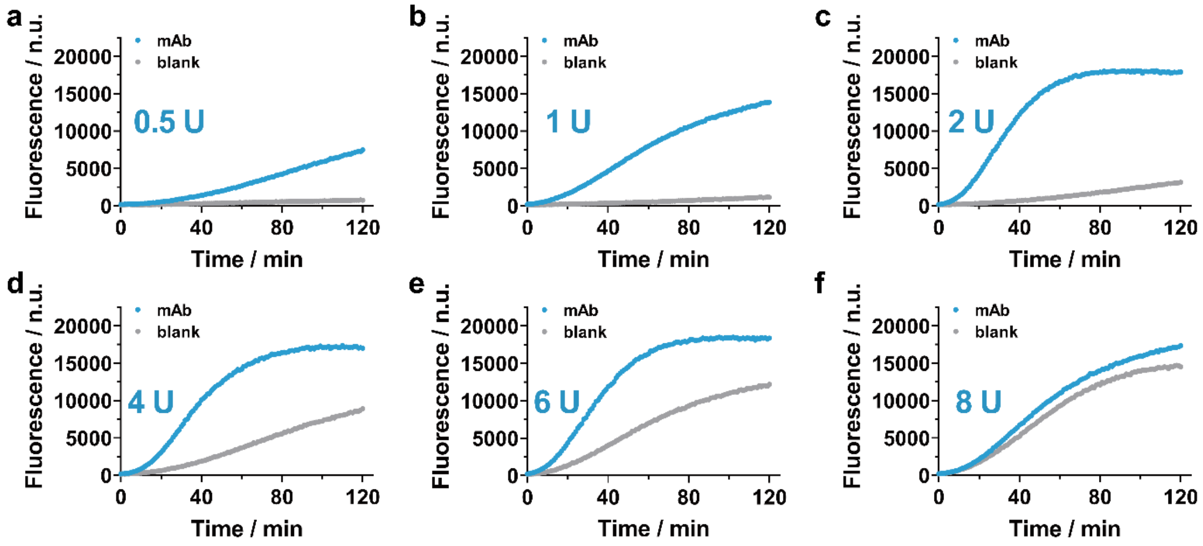

Supplementary Figure 2 | Optimization of T4 polymerase. Optimization of T4 polymerase from 0.5 U to 8 U per reaction in detecting anti-RBD mAb by UCAD. Based on the maximal differences between signal and background, an optimal concentration of T4 polymerase was found to be 2U per reaction. [mAb]= 100 fM, [TS probe] = 10 pM, [NTS probe] = 10 pM, [dNTPs]= 40 µM, [Cas12a] = 40 nM, [crRNA] = 40 nM, [Reporter] = 40 nM.

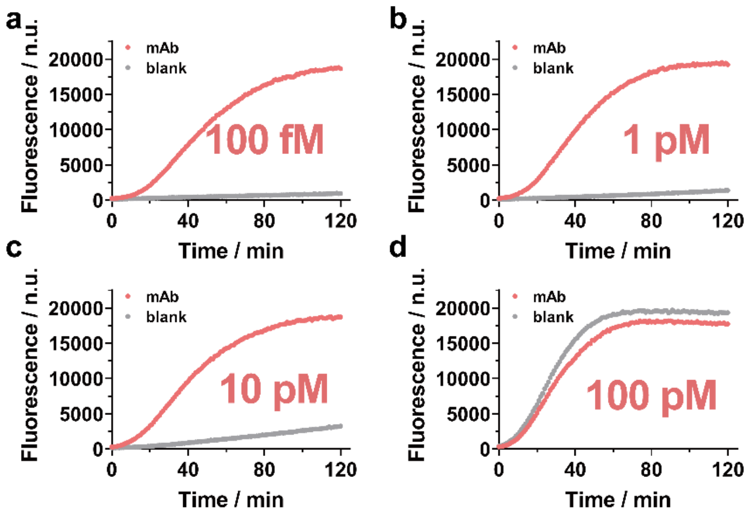

Supplementary Figure 3 | Optimization of UCAD probe concentration. Optimization of UCAD probe concentration in detecting anti-RBD mAb. Despite 1 pM TS and NTS probe concentration demonstrates the best signal to background ratio, 10 pM was chosen as the optimal TS and NTS probe concentration to maximize the dynamic range for the detection of anti-RBD mAb. [mAb] = 100 fM, [T4 polymerase] = 2 U, [dNTPs]= 40 µM, [Cas12a] = 40 nM, [crRNA] = 40 nM, [Reporter] = 40 nM.

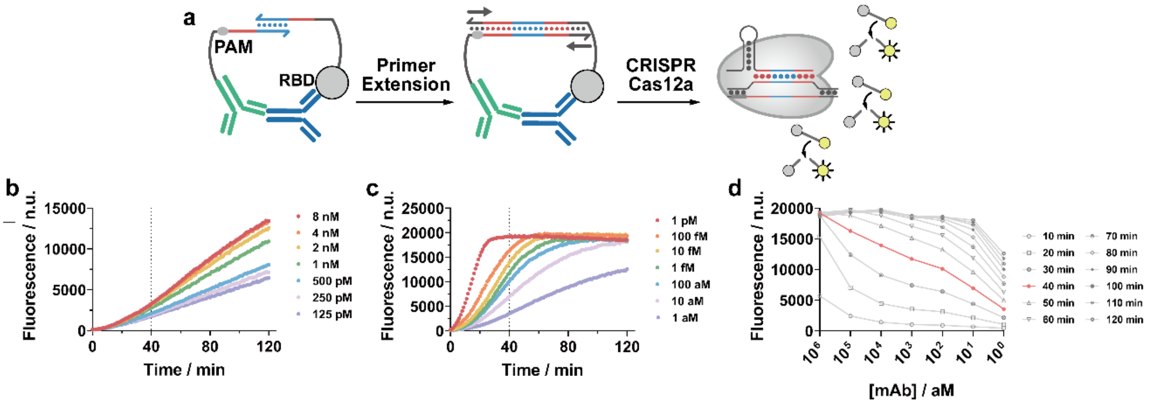

Supplementary Figure 4 | Sensitivity of UCAD in buffer. (**a**) Schematic illustration of UCAD workflow in detecting anti-RBD human mAb without nucleic acid amplification of the pre-designed dsDNA barcode. (**b**) Kinetic curves for mAb detection using UCAD without RPA. The concentrations of mAb were ranged from 125 pM to 8 nM. (**c**) Kinetic curves for mAb detection using UCAD with RPA. The concentration of mAb were ranged from 1 aM to 1 pM. [TS probe] = 10 pM, [NTS probe] = 10 pM, [T4 polymerase] = 2 U, [dNTPs]= 40 μM, [Cas12a] = 40 nM, [crRNA] = 40 nM, [FQ-labeled reporter] = 40 nM. (**d**) Calibration curves using end-point fluorescence signals of mAb ranged from 1 aM to 1 pM at different time points of UCAD reaction. The fluorescence signal at 40 min showed the best dynamic range and was thus selected as the best time point for subsequent experiments and data processing.

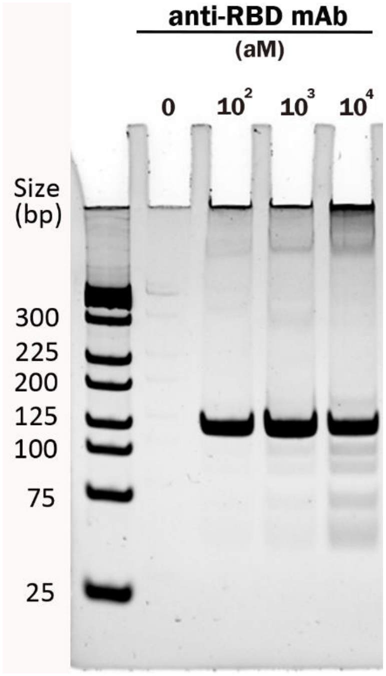

Supplementary Figure 5 | Native PAGE analysis of UCAD. Native PAGE analysis of the regeneration and RPA amplification of dsDNA barcode for UCAD. The optimal workflow for UCAD was used to regenerate and amplify dsDNA barcode in response to 100 aM to 10 fM anti-RBD mAb. RPA amplicon was analyzed using 12% PAGE gel. 1 μL of UCAD product was mixed with 5 μL loading buffer before loading on gel. After electrophoresing in 1× TAE buffer at 120 V for 1 h, the gel was stained and visualized using GenoSens 2000 (CLINX). From left to right: Lane 1: 25 – 500 bp DNA ladder, Lane 2: reaction buffer containing no anti-RBD mAb (Blank), Lane 3: reaction buffer containing 100 aM anti-RBD mAb, Lane 4: reaction buffer containing 1 fM anti-RBD mAb, Lane 5: reaction buffer containing 10 fM anti-RBD mAb. Reaction buffer: [TS probe] = 10 pM, [NTS probe] = 10 pM, [dNTPs]= 40 μM, [T4 polymerase] = 2 U. The experiment was repeated twice independently to get similar results.

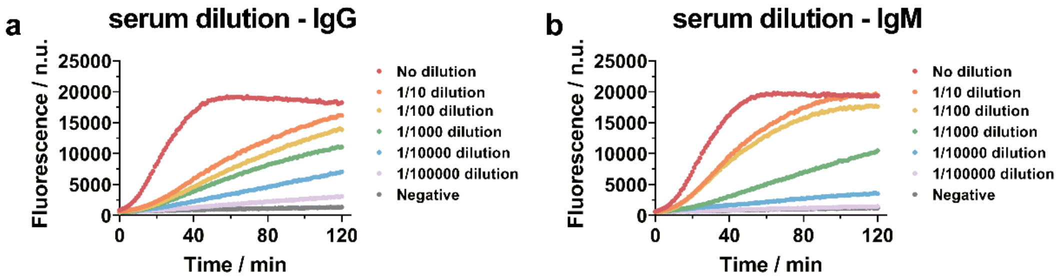

Supplementary Figure 6 | Sensitivity of UCAD in human serum samples. Detection of anti-RBD IgG (**a**) and IgM (**b**) in certified anti-SARS-CoV-2 positive human serum sample and diluted sera using certified COVID-19 negative human serum sample. [TS probe] = 10 pM, [NTS probe] = 10 pM, [T4 polymerase] = 2 U, [dNTPs]= 40 μM, [Cas12a] = 40 nM, [crRNA] = 40 nM, [FQ-labeled reporter] = 40 nM.

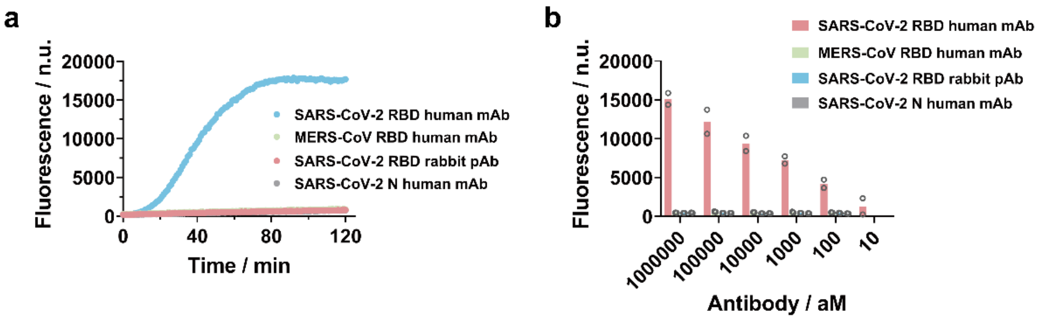

Supplementary Figure 7 | Specificity of UCAD. Evaluating the specificity of UCAD for anti-SARS-CoV-2 spike protein RBD human mAb (SARS-CoV-2 RBD human) against closely related anti- MERS-CoV spike protein RBD human mAb (MER-CoV RBD human), anti-SARS-CoV-2 spike protein RBD rabbit pAb (SARS-CoV-2 RBD rabbit) and anti-SARS-CoV-2 N nucleocapsid protein mAb (SARS-CoV-2 N human). (**a**) UCAD kinetic curves for 10 fM anti-SARS-CoV-2 RBD against equal concentrations of nonspecific antibodies. (**b**) Detection of SARS-CoV-2 RBD human mAb and nonspecific antibodies in the concentration range from 1 pM to 10 aM using UCAD. Only SARS-CoV-2 RBD human mAb generated fluorescence signal linearly correlated with its concentration (n = 2 biological independent samples). [TS probe] = 10 pM, [NTS probe] = 10 pM, [T4 polymerase] = 2 U, [dNTPs]= 40 μM, [Cas12a] = 40 nM, [crRNA] = 40 nM, [Reporter] = 40 nM.

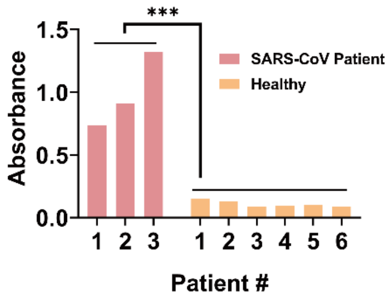

Supplementary Figure 8 | Validation of the SARS-CoV serum sample. A standard ELISA test was performed to validate the presence of anti-SARS-CoV IgG in three human serum samples from patients with confirmed SARS-CoV infections. Serum samples from six healthy individuals were also included to ensure the specificity of the ELISA test. From the unpaired two tailed t-test, the difference between the two cohorts were significant ($p = 0.0001$). ***, $p \leq 0.001$.

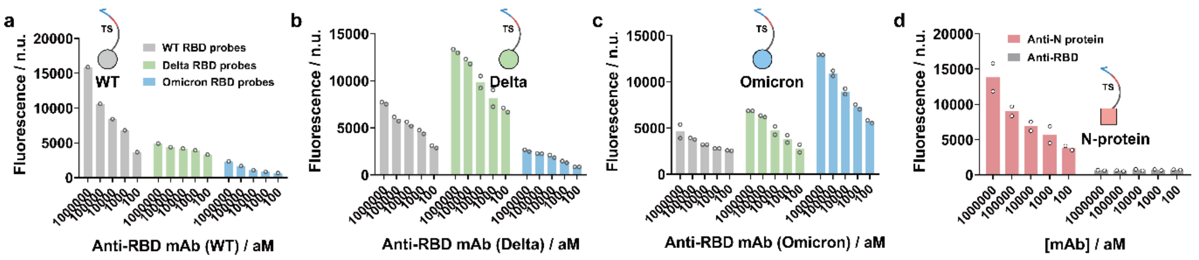

Supplementary Figure 9 | Modularity of UCAD. (**a**) Detection of human anti-RBD mAbs against wild-type (WT) SARS-CoV-2, Delta mutant, and Omicron mutant using a WT-specific TS probe. (**b**) Detection of human anti-RBD mAbs against wild-type (WT) SARS-CoV-2, Delta mutant, and Omicron mutant using a Delta-specific TS probe. (**c**) Detection of human anti-RBD mAbs against wild-type (WT) SARS-CoV-2, Delta mutant, and Omicron mutant using an Omicron-specific TS probe. (**d**) Detection of anti-SARS-CoV-2 nucleocapsid (N) human mAb (anti-N protein) and anti-RBD using N protein-specific TS probe. All antibodies were tested in a concentration range from 1 pM to 100 aM. All data were generated from 2 biological independent samples (n = 2). [TS probe] = 10 pM, [NTS probe] = 10 pM, [T4 polymerase] = 2 U, [dNTPs]= 40 µM, [Cas12a] = 40 nM, [crRNA] = 40 nM, [Reporter] = 40 nM.

S2. Demographic information for clinical validation of UCAD

Supplementary Table 2. Demographic characteristics of 85 KTRs.

	IgG^+/IgM^+ (n=73)	IgG^-/IgM^- (n=12)
Donor type		
Living	29(39.7%)	8(66.7%)
Deceased	44(60.3%)	4(33.3%)
Recipient gender		
Male	54(74.0%)	7(58.3%)
Female	19(26.0%)	5(41.7%)
Age (year)	41.9±11.1	40.3±7.2
Transplantation to vaccination (month)	114±73	119±88
Sample from vaccination (day)	29±14	28±17
Induction therapy		
No	6(8.2%)	2(16.7%)
ATG/ATGF	22(30.1%)	5(41.7%)
Basiliximab	45(61.6%)	5(41.7%)
Steroid pulse therapy in 12 months		
Yes	1(1.4%)	0
No	72(98.6%)	0
ATG (anti-thymocyte globulin therapy) in 12 months		
Yes	1(1.4%)	0
No	72(98.6%)	0
eGFR (estimated glomerular filtration rate) before vaccination (ml/min)	64±19	70±11
Albumin before vaccination (g/L)	45.5±3.4	46.8±2.8
Globulin before vaccination (g/L)	24.2±4.1	23.9±2.5
Hemoglobulin before vaccination (g/L)	143±19	140±17
WBC (white blood cell) before vaccination (10^9/L)	7.5±2.4	7.2±1.6
Neutrophil before vaccination (10^9/L)	4.9±1.9	4.6±1.1
Lymphocyte before vaccination (10^9/L)	1.9±0.7	1.9±0.5
Tacrolimus trough level before vaccination (ng/ml)	5.9±1.6	5.2±2.0

S3. UCAD and cellular analysis of KTRs

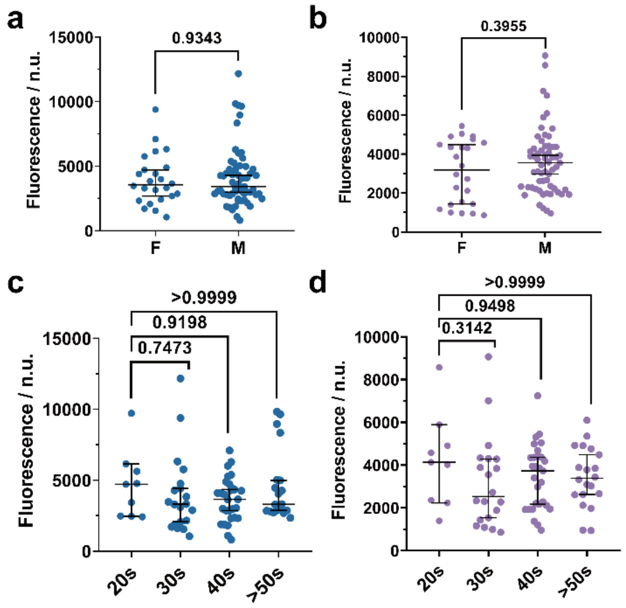

Supplementary Figure 10 | Correlation of anti-RBD level with gender and age. (a, b) Correlation of UCAD-determined anti-RBD IgG (**a**) and IgM (**b**) levels of 85 KTRs with gender. Female: n= 24 clinical independent samples, Male: n = 61 clinical independent samples. No significant differences in the levels of anti-RBD IgG (data are presented as 3941 ±1949 for females and 4129±2274 for males) and IgM (data are presented as 3071±1620 for females and 3601±1686 for males) were found between genders. (**c, d**) Correlation of the UCAD-determined anti-RBD IgG (**c**) and IgM (**d**) levels of 85 KTRs with age. No significant differences were found between varying age groups. 20s: n = 9 clinical independent samples, data are presented as 4643±2368 for IgG and 4231±2173 for IgM. 30s: n = 21 clinical independent samples, data are presented as 3954±2769 for IgG and 3189±2126 for IgM. 40s: n = 34 clinical independent samples, data are presented as 3645±1545 for IgG and 3430 ±1516 for IgM. 50s and over: n = 21 clinical independent samples,, data are presented as 4575±2504 for IgG and 3441±1395 for IgM. Comparison of anti-RBD IgG and IgM levels between two gender groups were performed using unpaired two tailed t-test. Nonparametric

Kruskal-Wallis test was used to evaluate the differences of the IgG and IgM levels between four age groups. Error bars represent standard deviation.

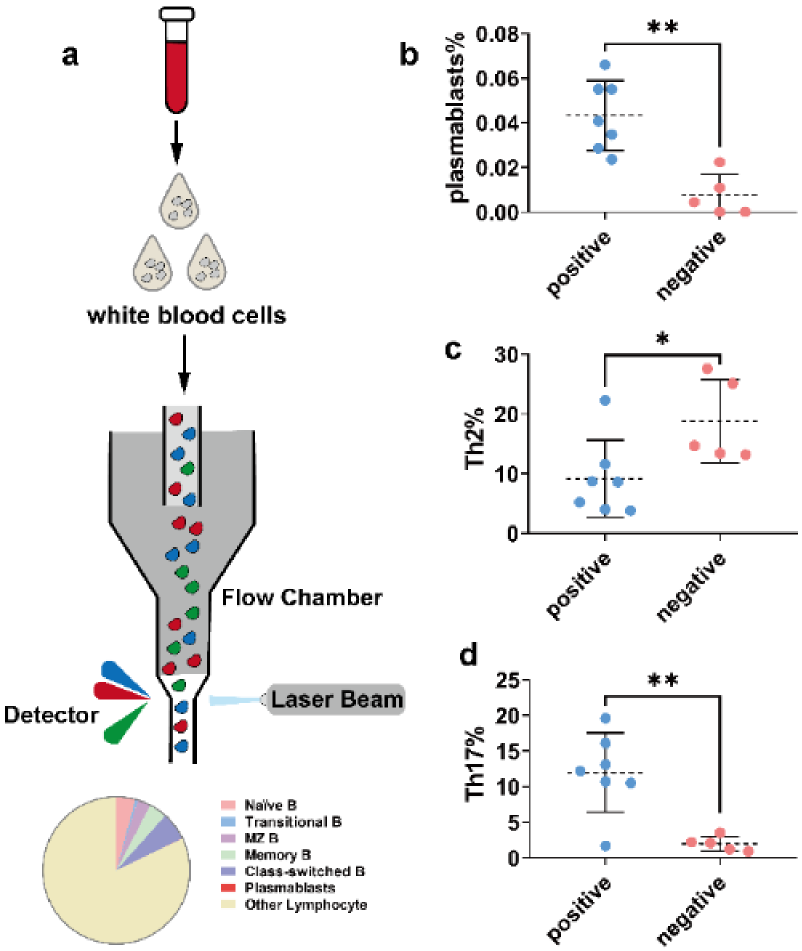

Supplementary Figure 11 | Flow Cytometric analysis of KTRs. Blood samples from seven anti-RBD positive vaccinated KTRs (P1, P2, P3, P4, P5, P6, P7), five anti-RBD negative KTRs (N1, N2, N3, N4, N5), as well as five anti-RBD positive sera from vaccinated healthy participates (H1, H2, H3, H4, H5) were analyzed using flow cytometry to determine the distribution of B and Th cell subsets. (**a**) Schematic illustration of flow cytometric analysis of white blood cells (WBCs) in UCAD-determined anti-RBD positive and negative KTRs (**b**) The percentage composition of plasmablasts in lymphocytes of 7 randomly chosen IgG^{+}/IgM^{+} KTRs (n = 7 clinical independent samples) and 5 randomly chosen IgG^{-}/IgM^{-} KTRs (n = 5 clinical independent samples). Unpaired two tailed t-tests showed that the IgG^{-}/IgM^{-} KTR cohort had significantly lower plasmablast levels than the IgG^{+}/IgM^{+} KTR cohort (p = 0.0014). Data are presented as 0.04338±0.01567 for IgG^{+}/IgM^{+} and 0.007520±

0.009421 for IgG^-/IgM^- KTRs. (**c**) The percentage composition of Th2 cells in $CD4^+$ cells of the 7 IgG^+/IgM^+ (n = 7 clinical independent samples) and 5 IgG^-/IgM^- KTRs (n = 5 clinical independent samples). The IgG^-/IgM^- KTR cohort had mildly elevated Th2 levels (p = 0.0289) compared with the IgG^+/IgM^+ KTR cohort. Data are presented as 9.171 ± 6.457 for IgG^+/IgM^+ and 18.80 ± 6.972 for IgG^-/IgM^- KTRs. (**d**) The percentage composition of Th17 cells in $CD4^+$ cells of the 7 IgG^+/IgM^+ (n = 7 clinical independent samples) and 5 IgG^-/IgM^- KTRs (n = 5 clinical independent samples). The IgG^-/IgM^- KTR cohort had substantially reduced Th17 levels (p = 0.0016) compared with the IgG^+/IgM^+ KTR group. Data are presented as 11.99 ± 5.565 for IgG^+/IgM^+ and 1.980 ± 1.018 for IgG^-/IgM^- KTRs . *: $p \leq 0.05$, **: $p \leq 0.01$. Error bars represent standard deviation. Raw Flow cytometry data for this Figure can be found in Supplementary Figure 14-25.

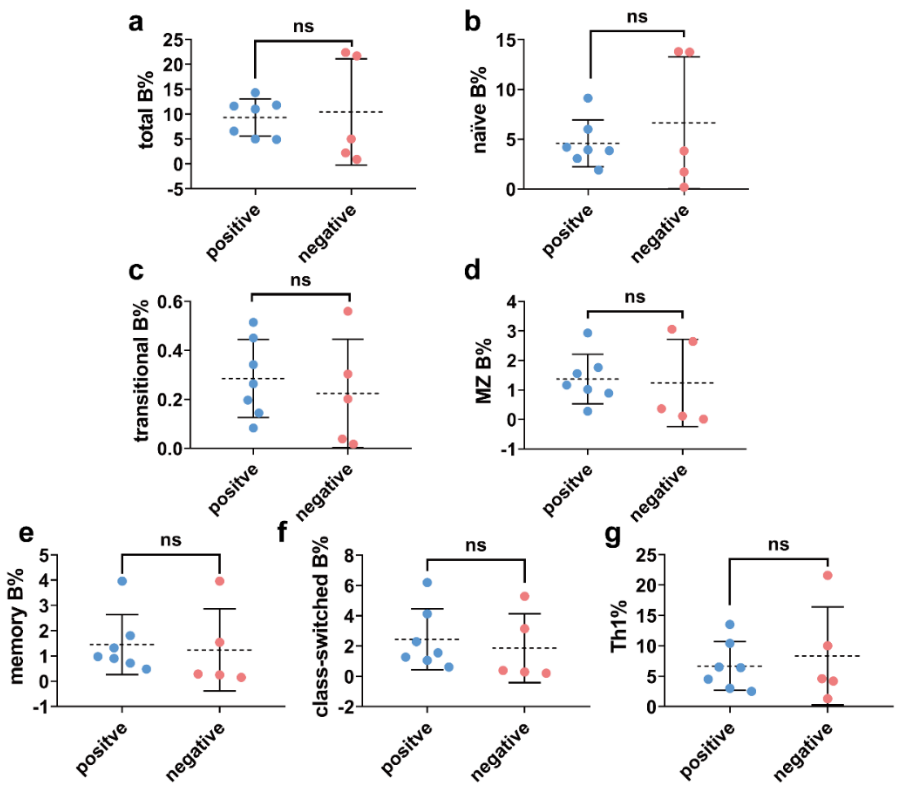

Supplementary Figure 12 | Anti-RBD positive *v.s.* negative KTRs. (**a-f**) The percentage composition of total B cell (**a**, p = 0.9506, data are presented as 9.314±3.754 for IgG^+/IgM^+ and 10.44±10.70 for IgG^-/IgM^- KTRs) and five B cell subsets: naïve B (**b**, p = 0.6570, data are presented as 4.585±2.355 for IgG^+/IgM^+ and 6.659±6.625 for IgG^-/IgM^- KTRs), transitional B (**c**, p = 0.8696, data are presented as 0.2854±0.1592 for IgG^+/IgM^+ and 0.2243 ±0.2218 for IgG^-/IgM^- KTRs), marginal zone (MZ) B (**d**, p = 0.9711, data are presented as 1.372±0.8384 for IgG^+/IgM^+ and 1.239±1.485 for IgG^-/IgM^- KTRs), memory B (**e**, p = 0.9482, data are presented as 1.452±1.187 for IgG^+/IgM^+ and 1.239±1.627 for IgG^-/IgM^- KTRs) and class-switched B (**f**, p = 0. 8497, data are presented as 2.444±2.015 for IgG^+/IgM^+ and 1.864±2.278 for IgG^-/IgM^- KTRs) in lymphocyte of seven IgG^+/IgM^+ KTRs (n = 7 clinical independent samples) and five IgG^-/IgM^- KTRs (n = 5 clinical independent samples). Unpaired two tailed t-tests showed no statistically significant differences of these variables between the two cohorts (p > 0.05). (**g**) The percentage compositive of Th1 in $CD4^+$ cells of the seven

IgG^+/IgM^+ KTRs (n = 7 clinical independent samples) and five IgG^-/IgM^- KTRs (n = 5 clinical independent samples). The two cohorts had no statistically significant difference in Th1 levels ($p = 0.8484$). Data are presented as 6.686 ± 4.005 for IgG^+/IgM^+ and 8.340 ± 8.051 for IgG^-/IgM^- KTRs. ns: $p > 0.05$. Error bars represent standard deviation. Raw Flow cytometry data for this Figure can be found in Supplementary Figure 14-25.

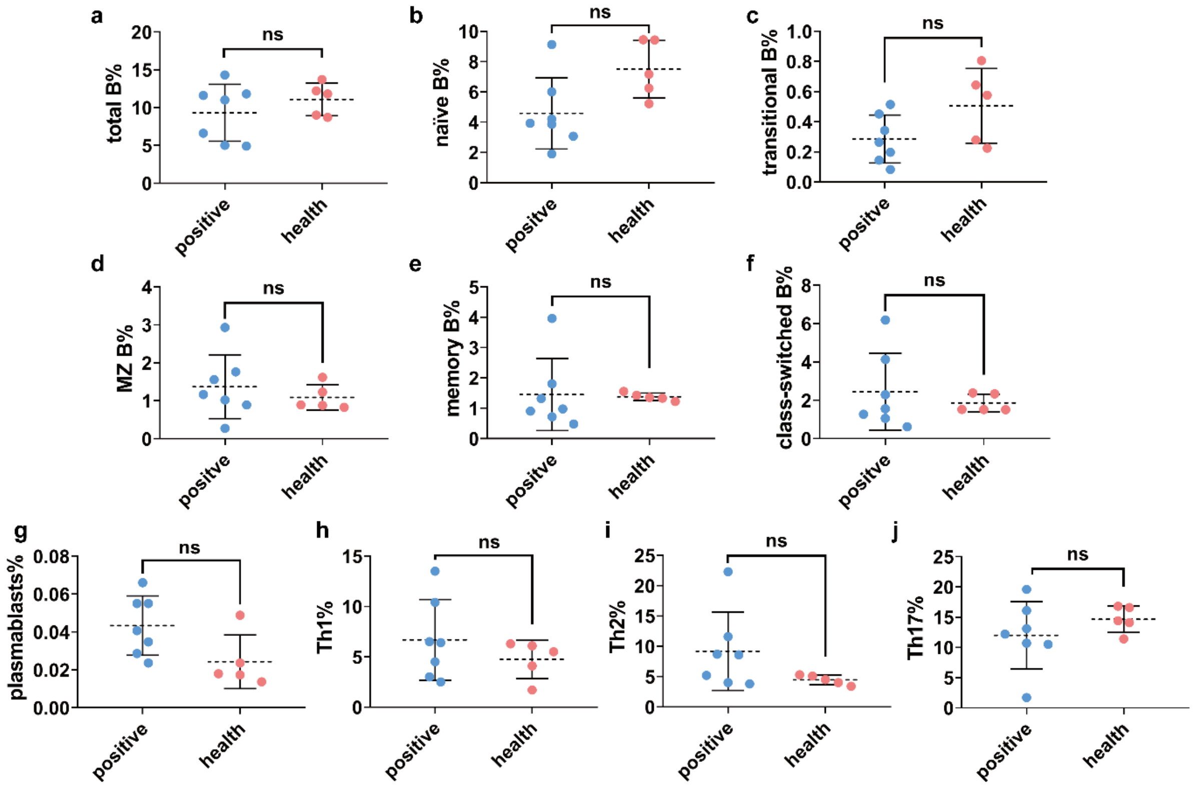

Supplementary Figure 13 | Anti-RBD positive KTRs *v.s.* anti-RBD positive health participates. (**a-g**) The percentage composition of total B cell (**a**, p = 0.8835, data are presented as 9.314±3.754 for IgG+/IgM+ KTRs and 11.08±2.158 for IgG+/IgM+ healthy participates) and five B cell subsets: naïve B (**b**, p = 0.4463, data are presented as 4.585±2.355 for IgG+/IgM+ KTRs and 7.502±1.895 for IgG+/IgM+ healthy participates), transitional B (**c**, p = 0.1971, data are presented as 0.2854±0.1592 for IgG+/IgM+ KTRs and 0.5055±0.2473 for IgG+/IgM+ healthy participates), marginal zone (MZ) B (**d**, p = 0.8756, data are presented as 1.372±0.8384 for IgG+/IgM+ KTRs and 1.088±0.3360 for IgG+/IgM+ healthy participates), memory B (**e**, p = 0.9935, data are presented as 1.452±1.187 for IgG+/IgM+ KTRs and 1.379 ±0.1236 for IgG+/IgM+ healthy participates), class-switched B (**f**, p = 0.8452, data are presented as 2.444±2.015 for IgG+/IgM+ KTRs and 1.854±0.4622 for IgG+/IgM+ healthy participates) and plasmablasts (**g**, p = 0.0773, data are presented as 0.04338±0.01567 for

IgG^+/IgM^+ KTRs and 0.02430 ± 0.01415 for IgG^+/IgM^+ healthy participates) in lymphocyte of seven IgG^+/IgM^+ KTRs (n = 7 clinical independent samples) and five IgG^+/IgM^+ healthy participates (n = 5 clinical independent samples). (**h-j**) The percentage compositive of Th1 (**h**, p = 0. 7974, data are presented as 6.686 ± 4.005 for IgG^+/IgM^+ KTRs and 4.740 ± 1.905 for IgG^+/IgM^+ healthy participates), Th2 (**i**, p = 0. 3559, data are presented as 9.171 ± 6.457 for IgG^+/IgM^+ KTRs and 4.460 ± 0.7829 for IgG^+/IgM^+ healthy participates), and Th17 (**j**, p = 0. 4831, data are presented as 11.99 ± 5.565 for IgG^+/IgM^+ KTRs and 14.66 ± 2.200 for IgG^+/IgM^+ healthy participates) in $CD4^+$ cells of the seven IgG^+/IgM^+ KTRs (n = 7 clinical independent samples) and five IgG^+/IgM^+ healthy participates (n = 5 clinical independent samples). Unpaired two tailed t-tests showed no statistically significant differences of these variables between the two cohorts ($p > 0.05$). ns: $p > 0.05$. Error bars represent standard deviation. Raw Flow cytometry data for this Figure can be found in Supplementary Figure 14-20 and Supplementary Figure 26-30.

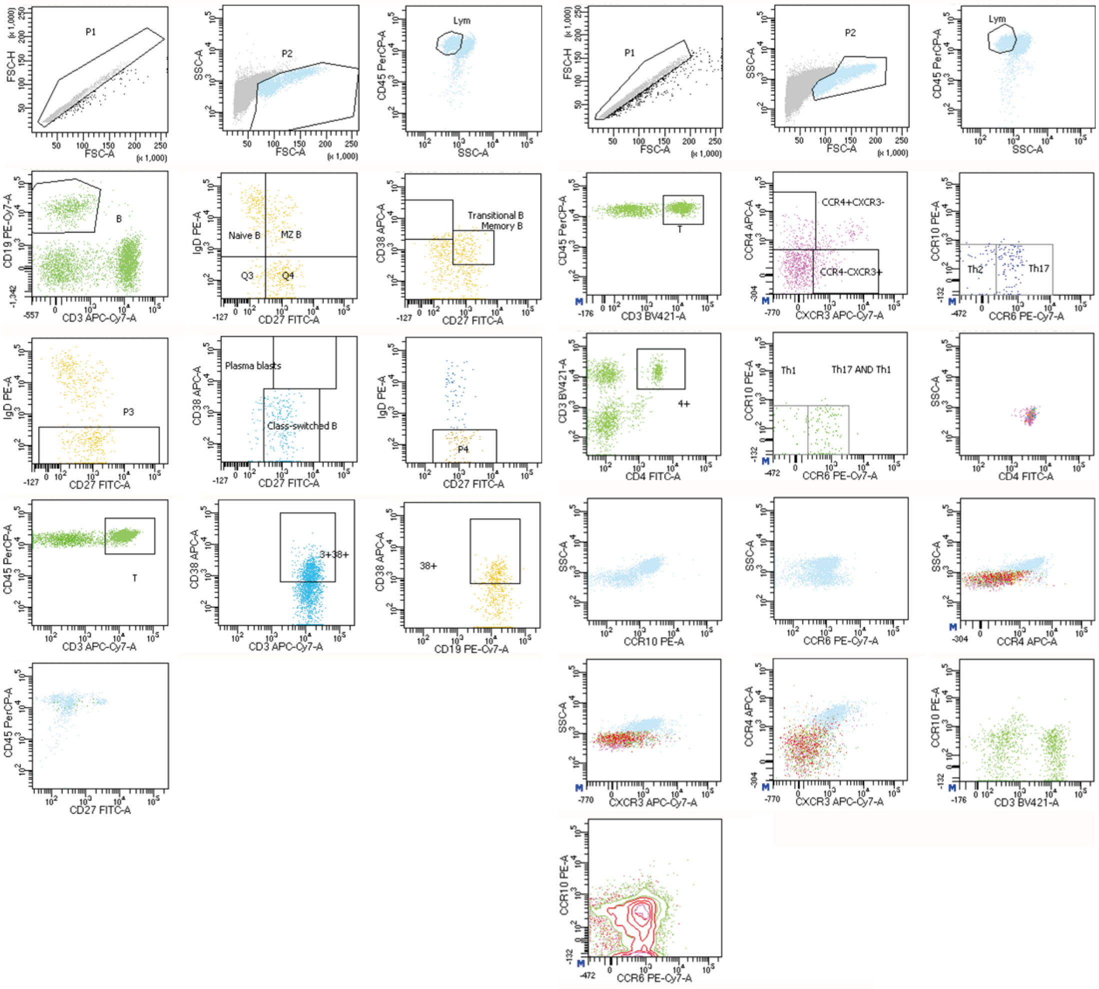

Supplementary Figure 14 | Flow cytometry analysis of B cell and Th cell subsets in patient sample P1.

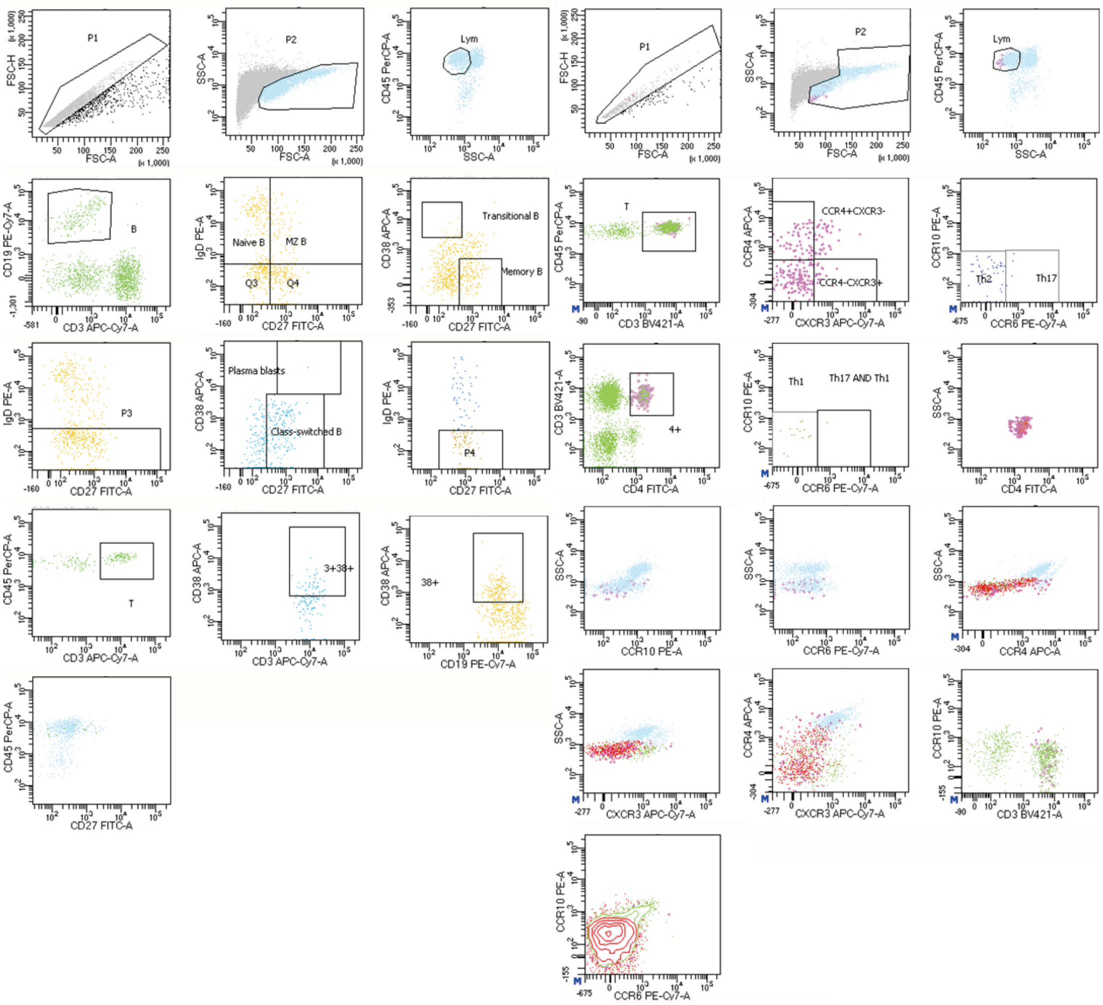

Supplementary Figure 15 | Flow cytometry analysis of B cell and Th cell subsets in patient sample P2.

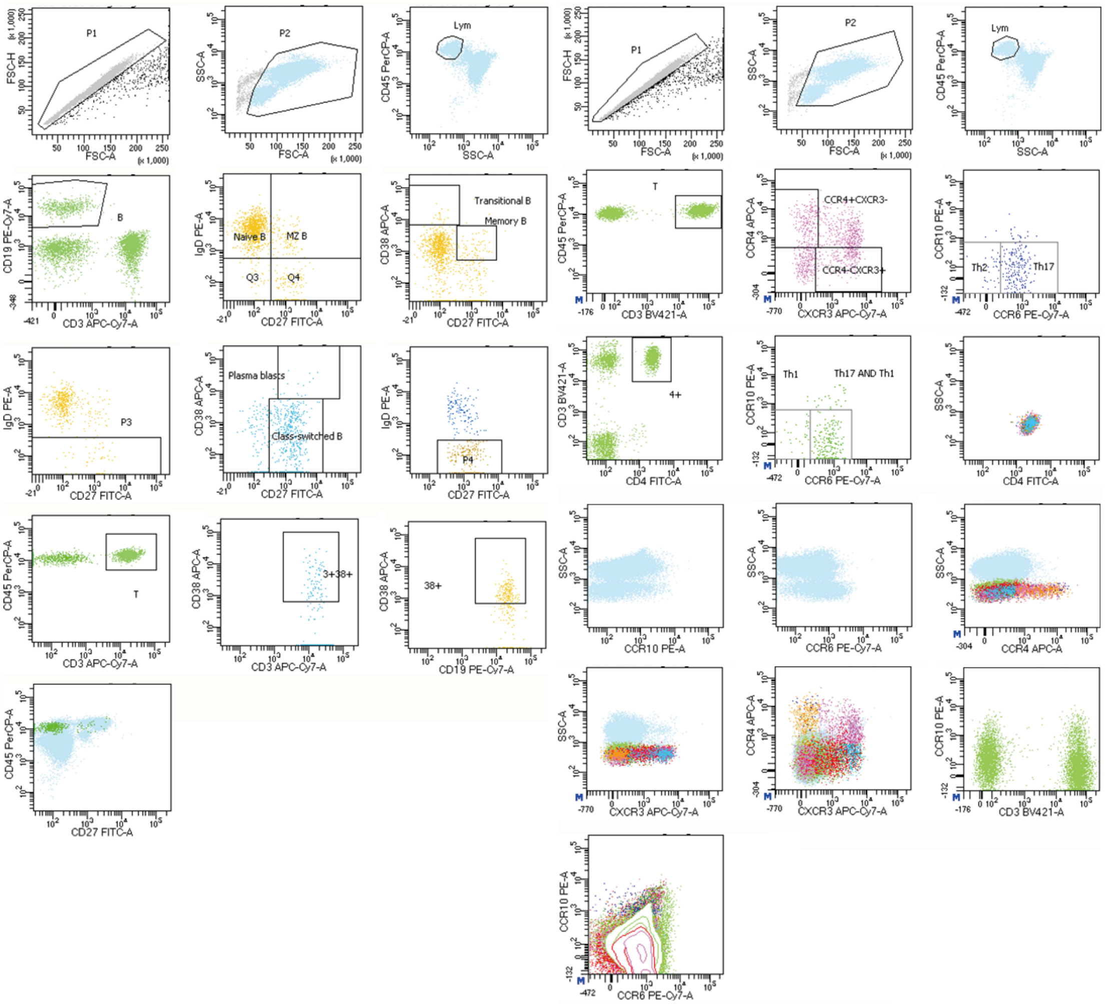

Supplementary Figure 16 | Flow cytometry analysis of B cell and Th cell subsets in patient sample P3.

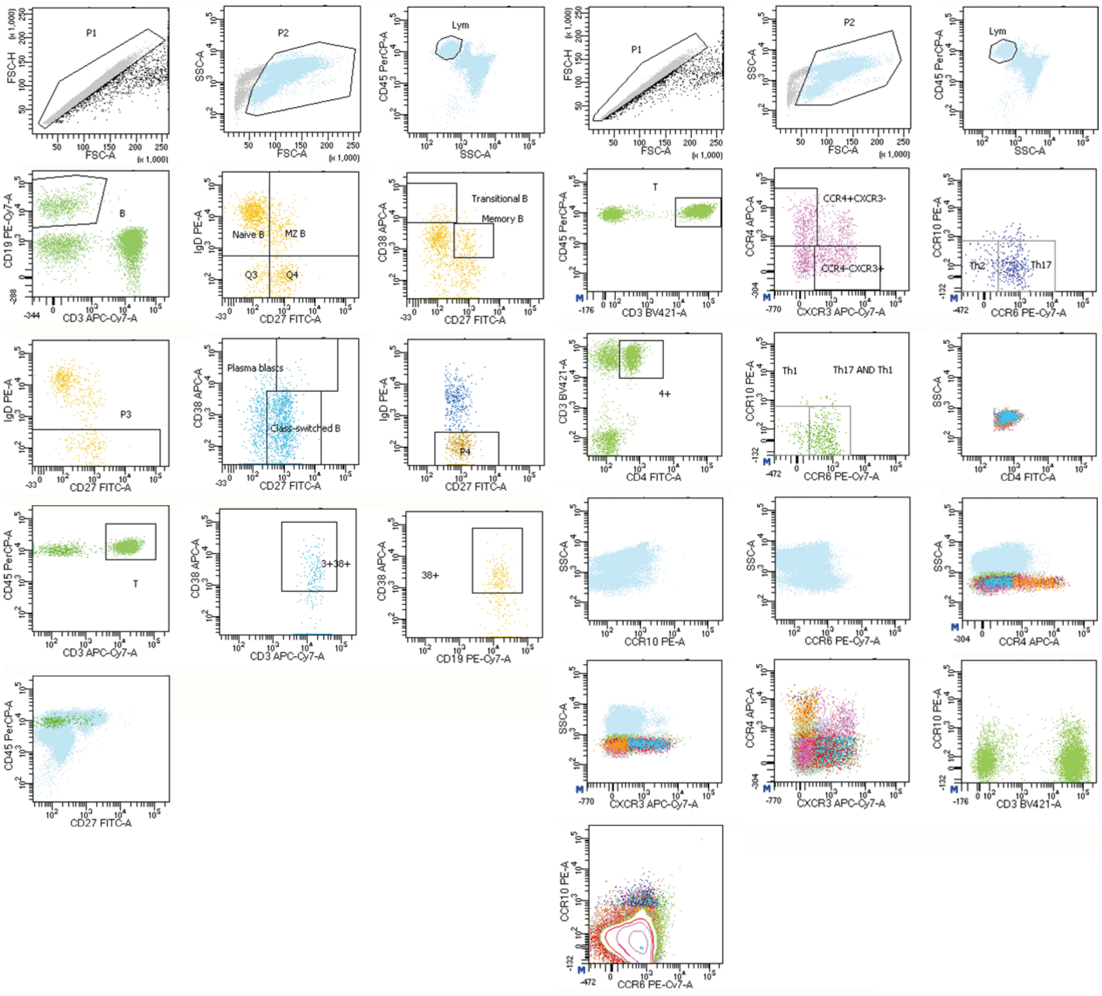

Supplementary Figure 17 | Flow cytometry analysis of B cell and Th cell subsets in patient sample P4.

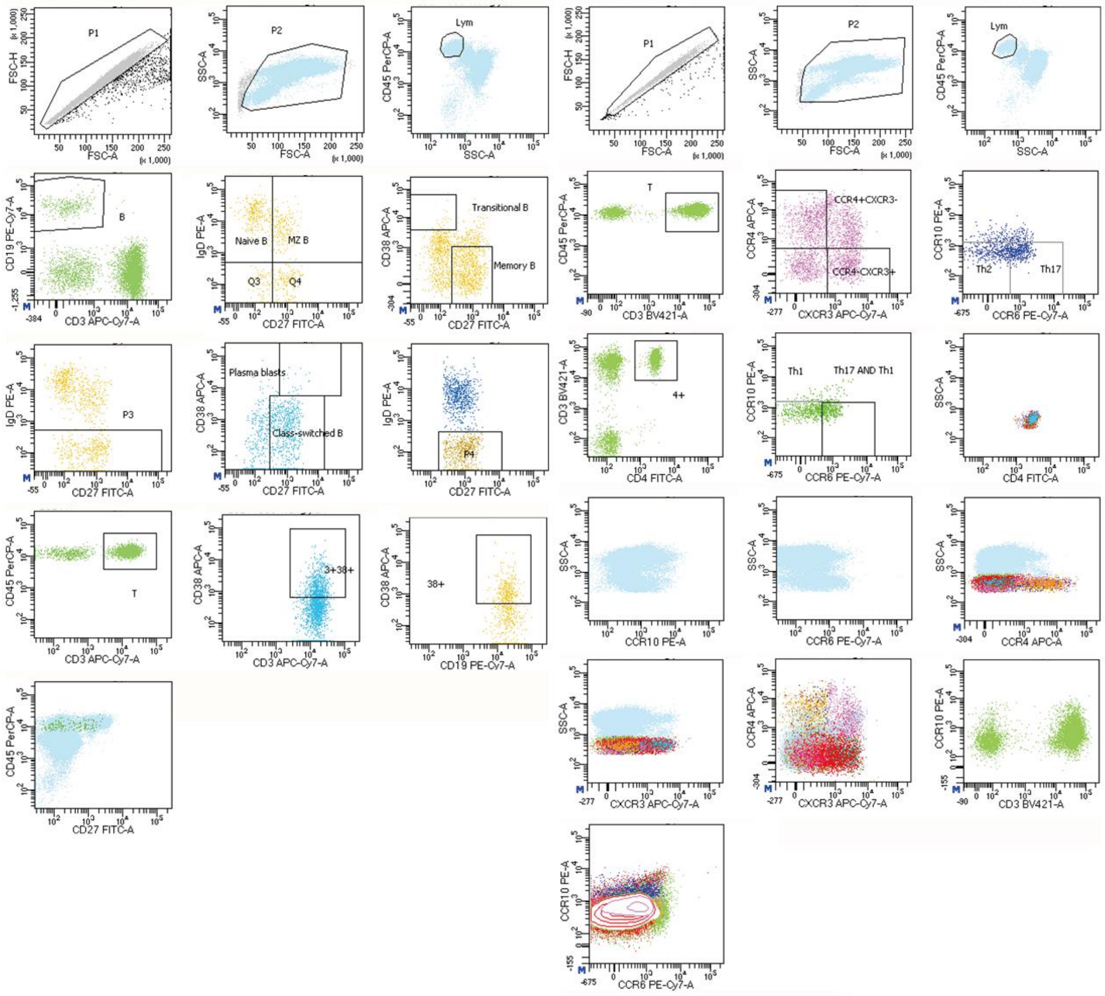

Supplementary Figure 18 | Flow cytometry analysis of B cell and Th cell subsets in patient sample P5.

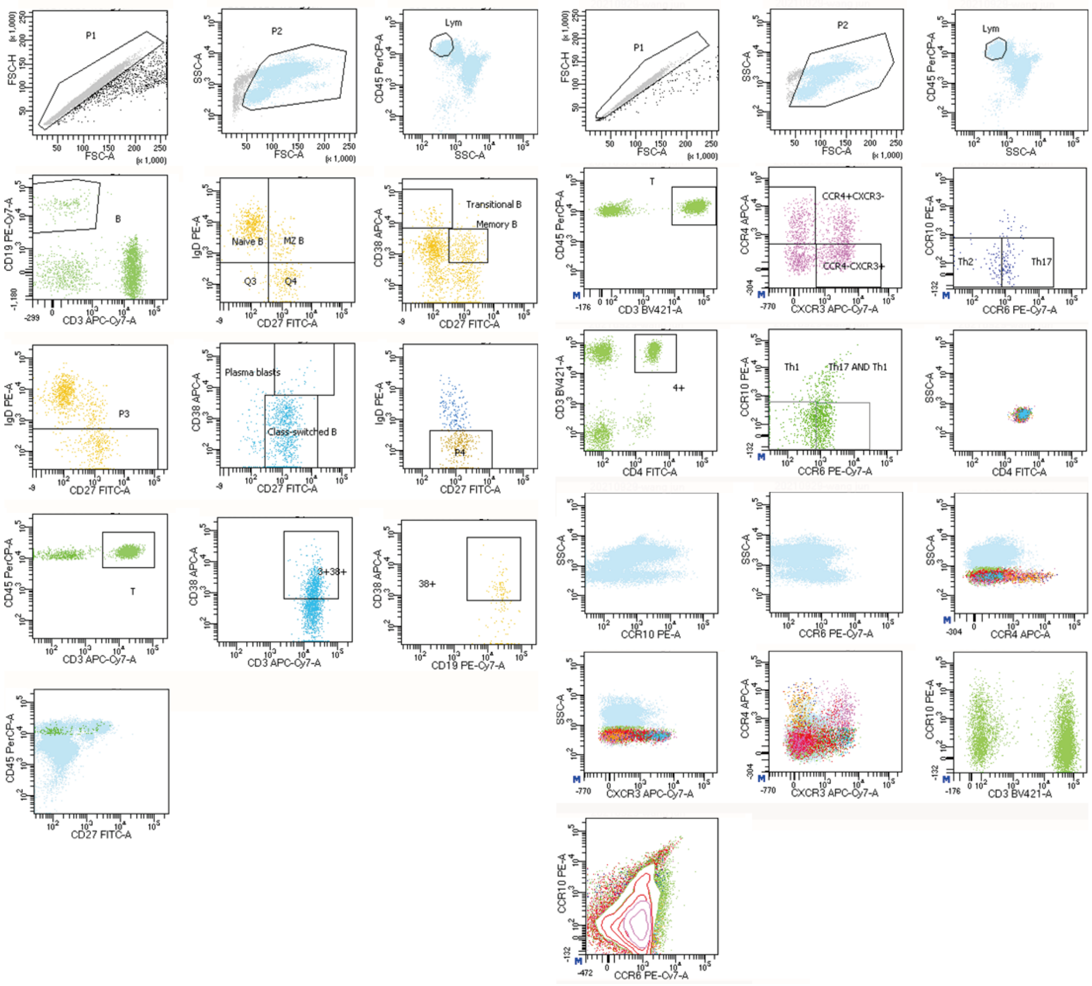

Supplementary Figure 19 | Flow cytometry analysis of B cell and Th cell subsets in patient sample P6.

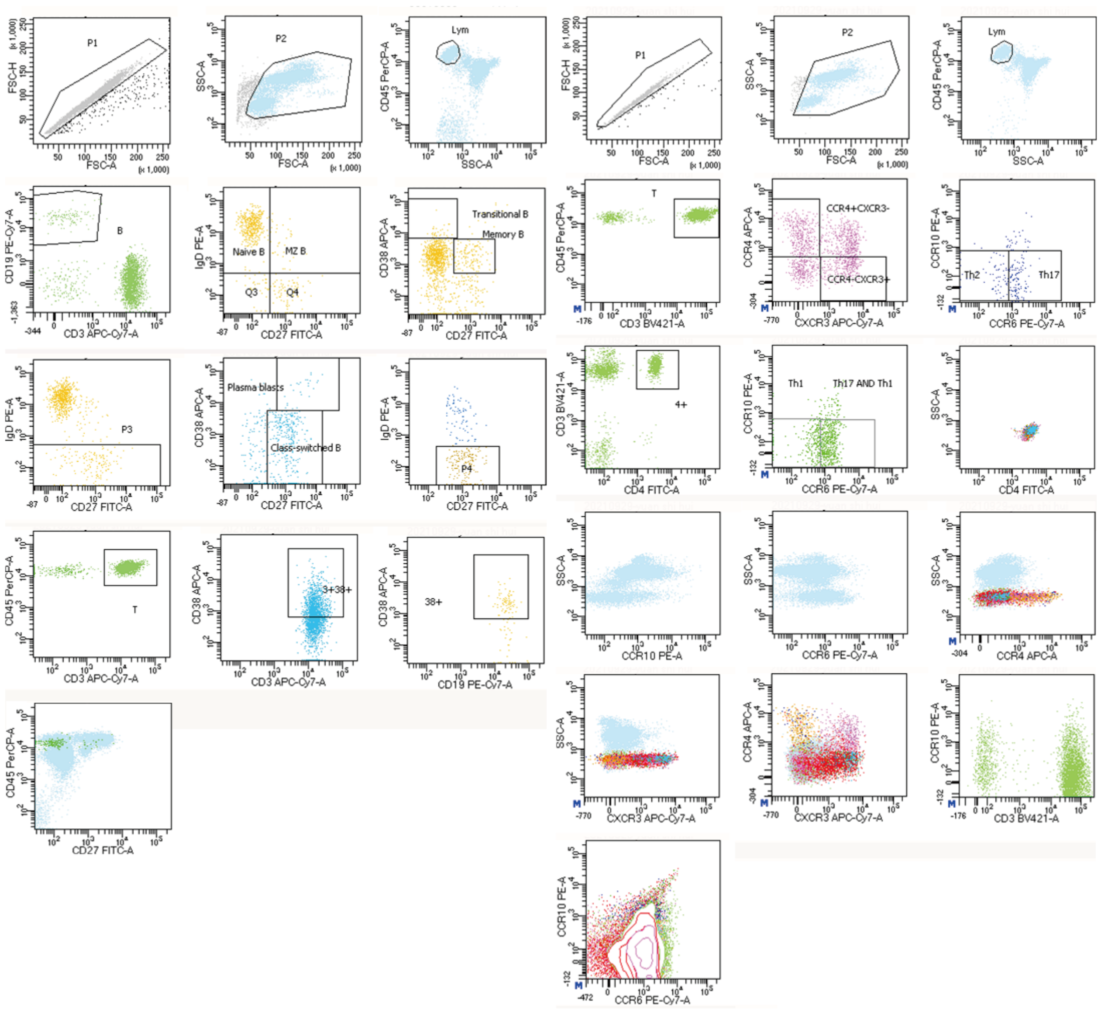

Supplementary Figure 20 | Flow cytometry analysis of B cell and Th cell subsets in patient sample P7.

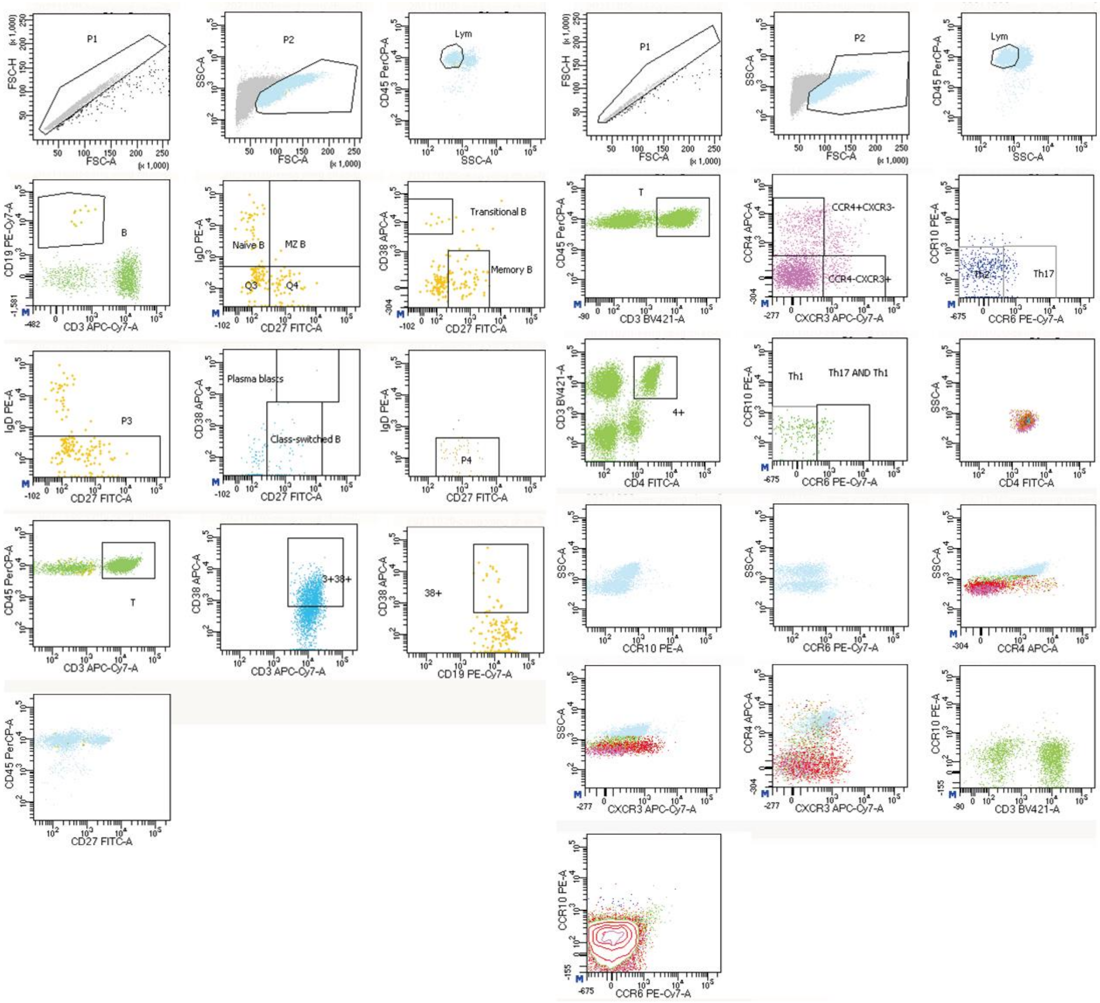

Supplementary Figure 21 | Flow cytometry analysis of B cell and Th cell subsets in patient sample N1.

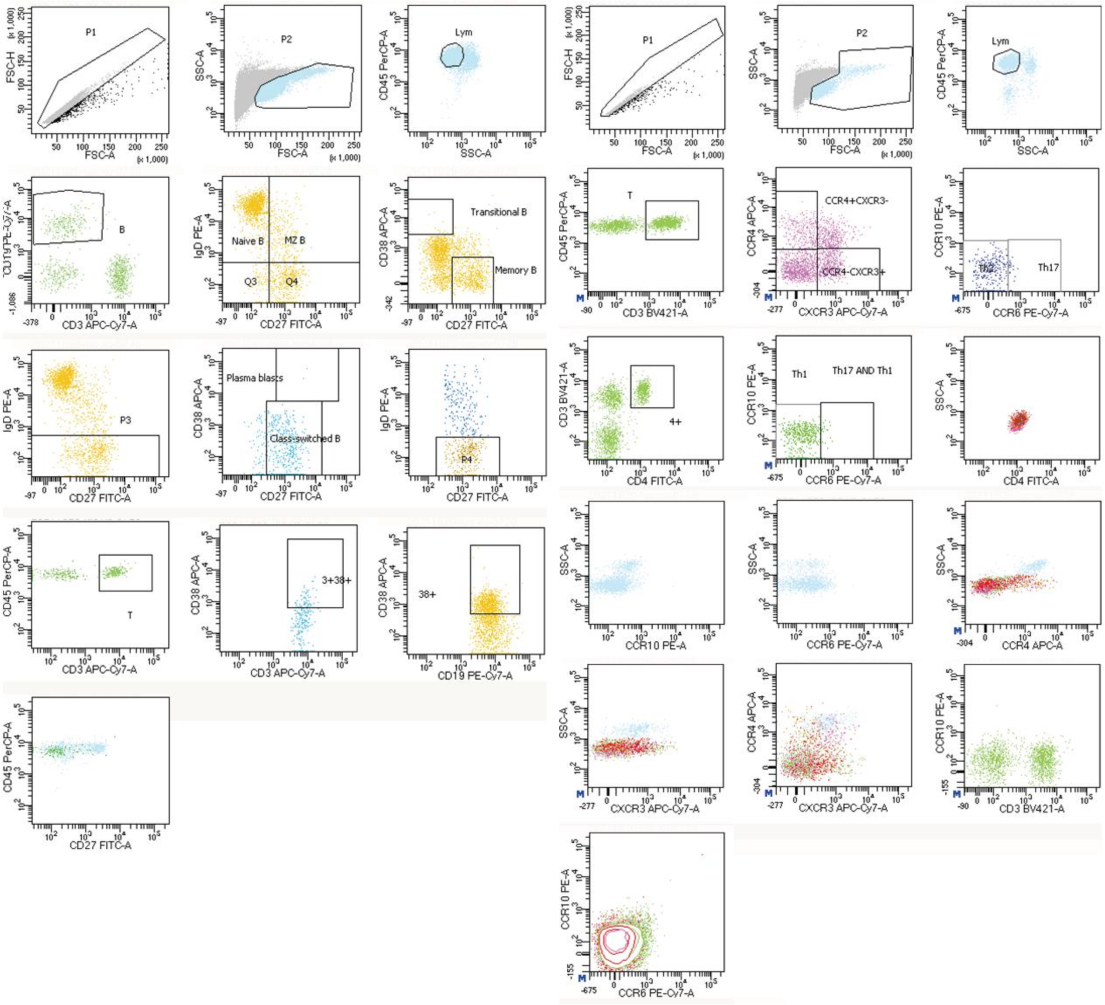

Supplementary Figure 22 | Flow cytometry analysis of B cell and Th cell subsets in patient sample N2.

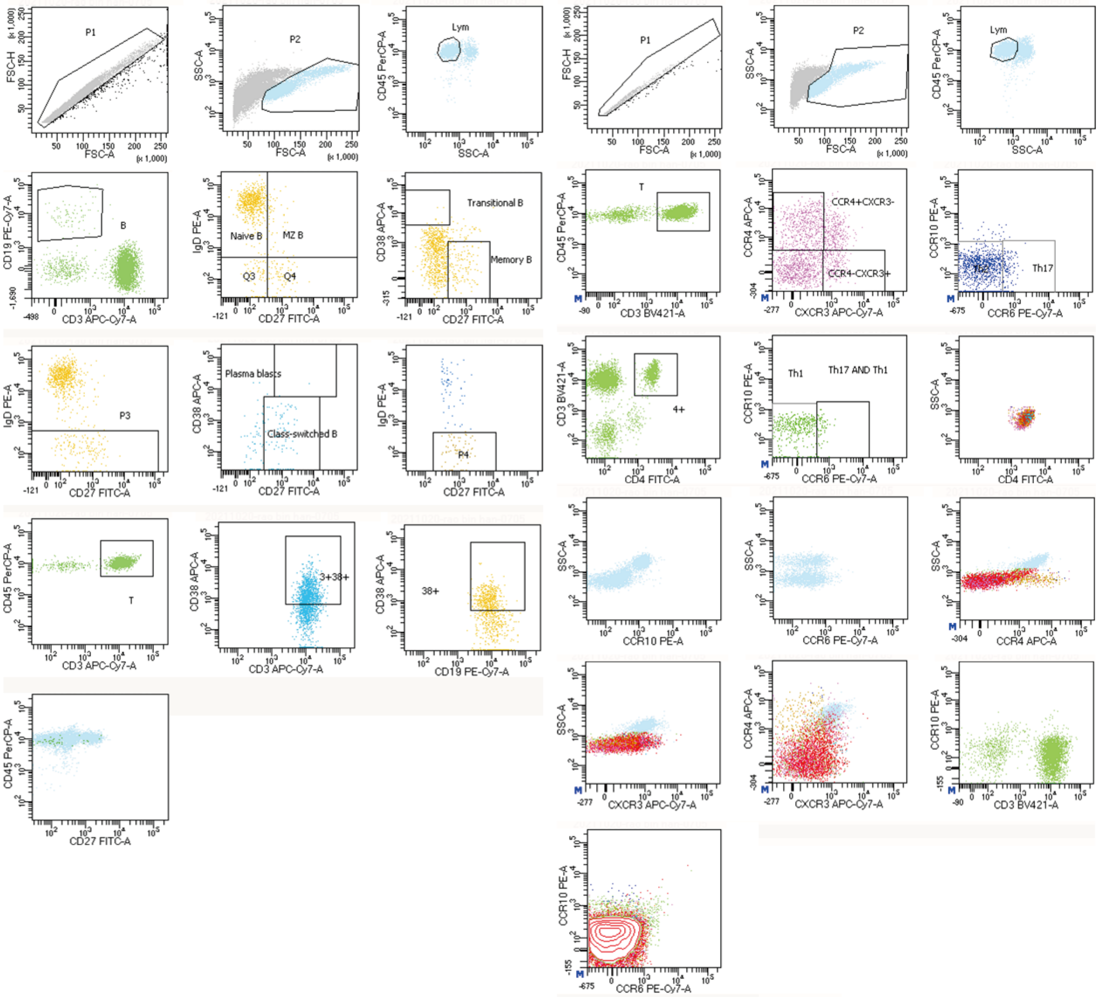

Supplementary Figure 23 | Flow cytometry analysis of B cell and Th cell subsets in patient sample N3.

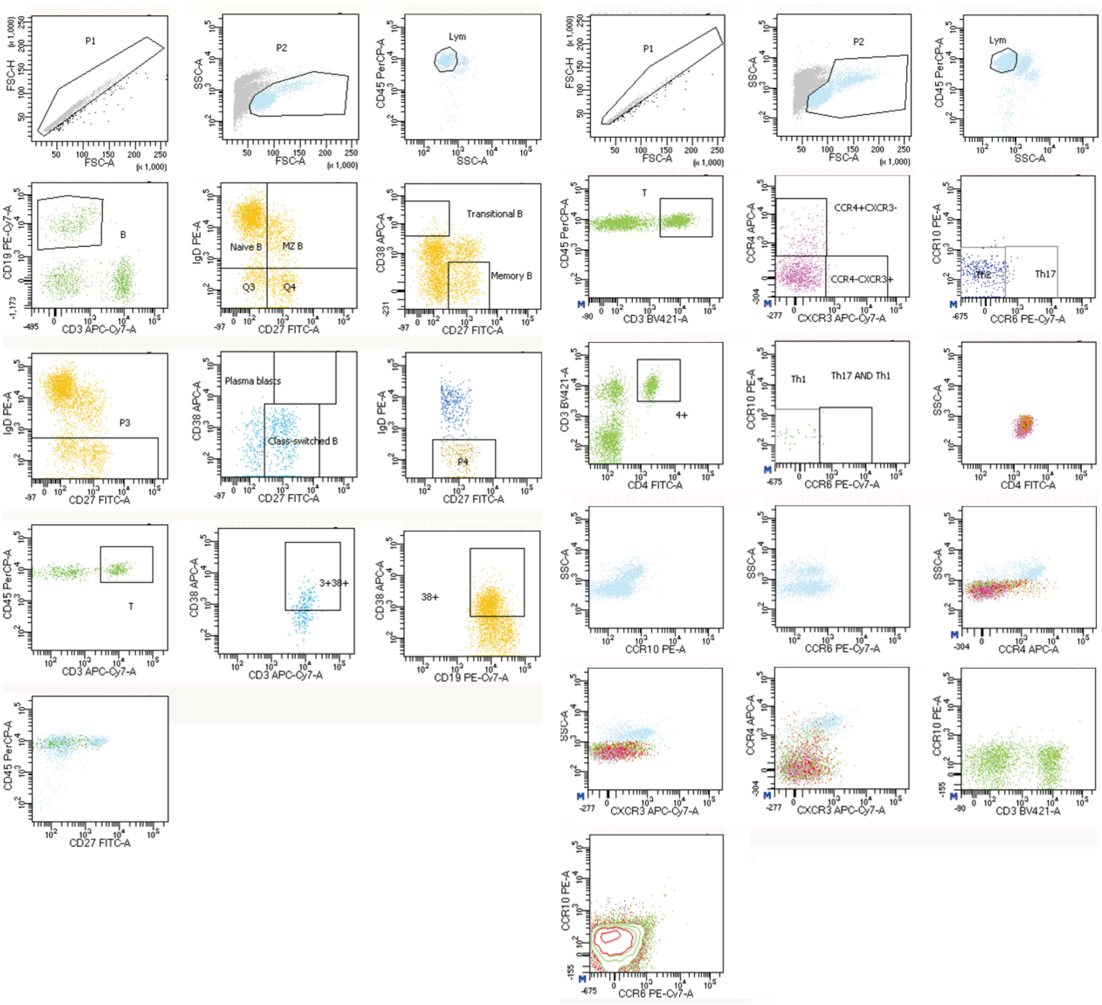

Supplementary Figure 24 | Flow cytometry analysis of B cell and Th cell subsets in patient sample N4.

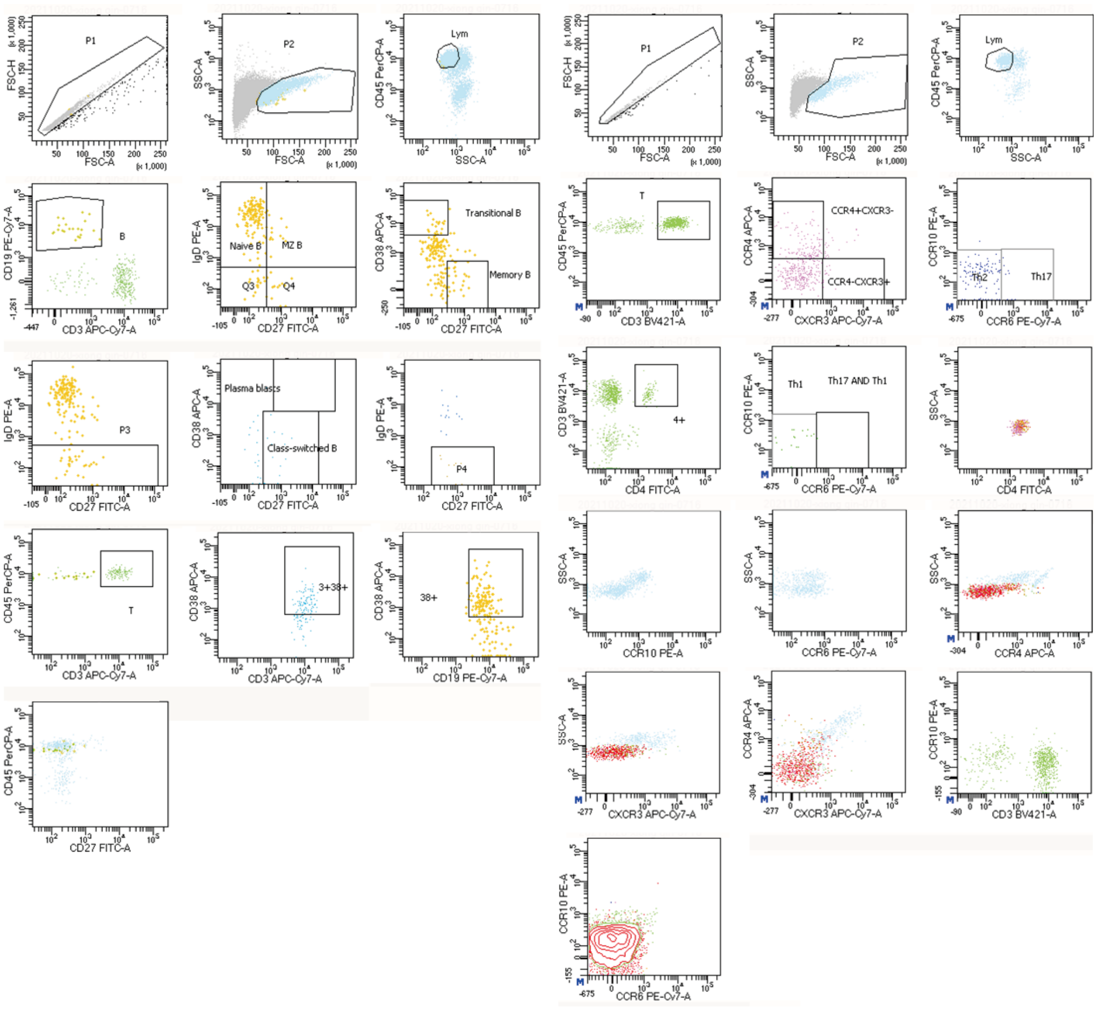

Supplementary Figure 25 | Flow cytometry analysis of B cell and Th cell subsets in patient sample N5.

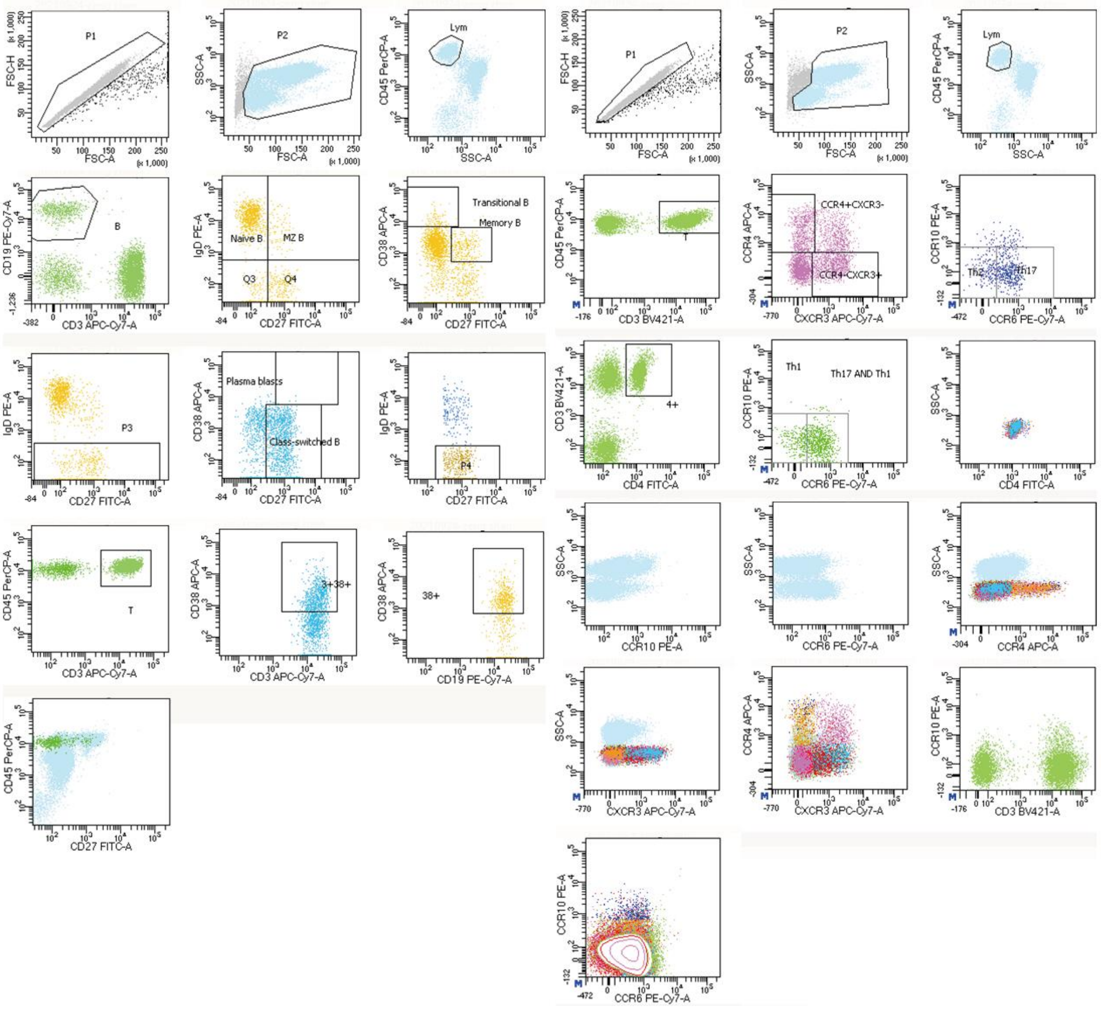

Supplementary Figure 26 | Flow cytometry analysis of B cell and Th cell subsets in patient sample H1.

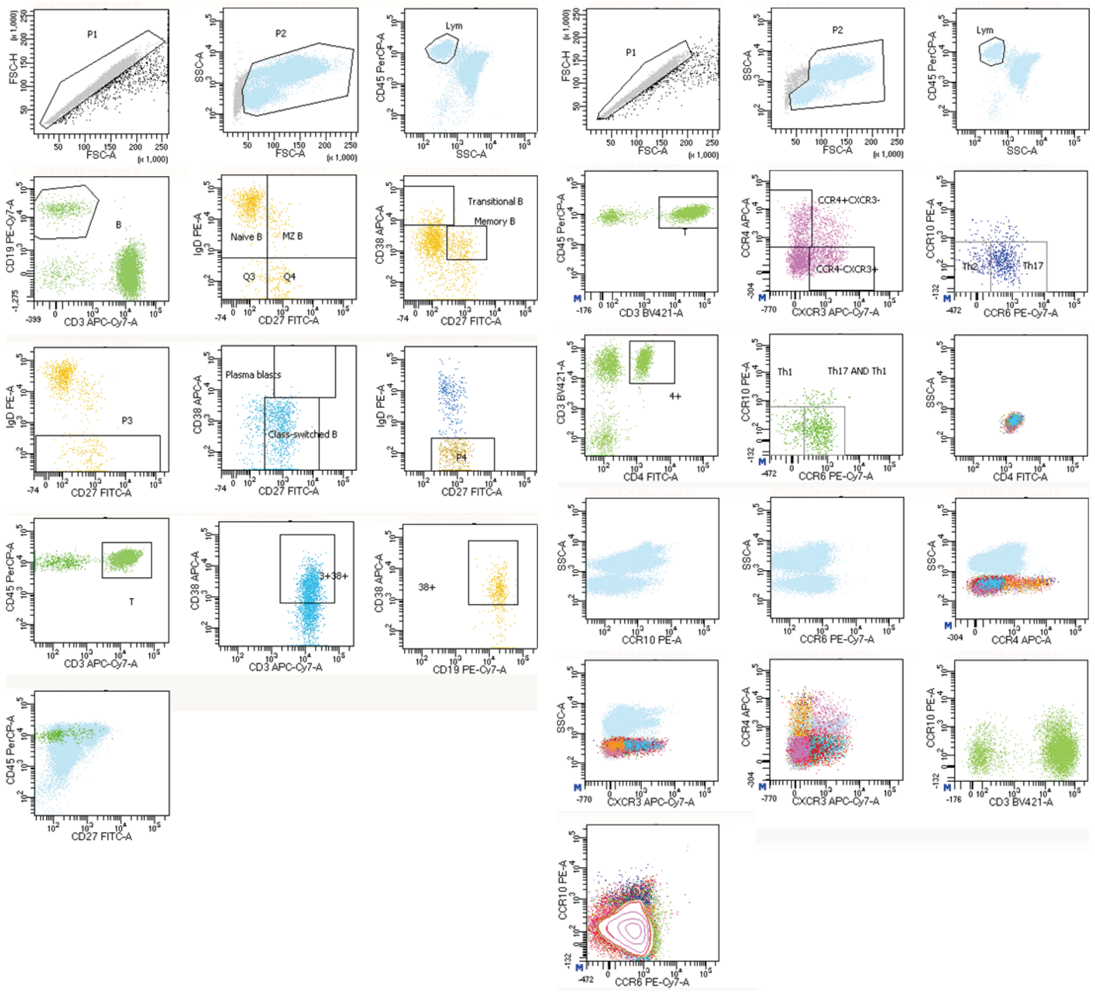

Supplementary Figure 27 | Flow cytometry analysis of B cell and Th cell subsets in patient sample H2.

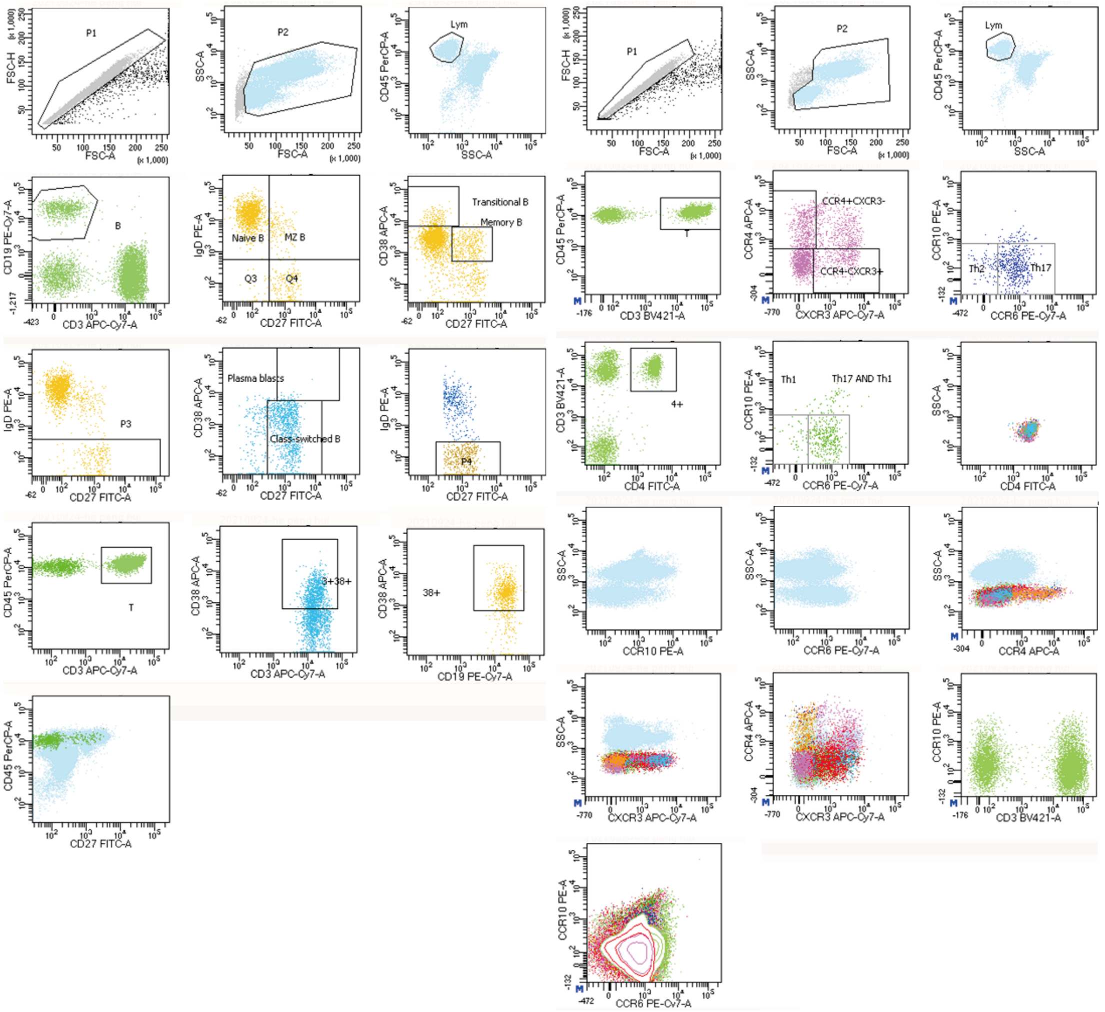

Supplementary Figure 28 | Flow cytometry analysis of B cell and Th cell subsets in patient sample H3.

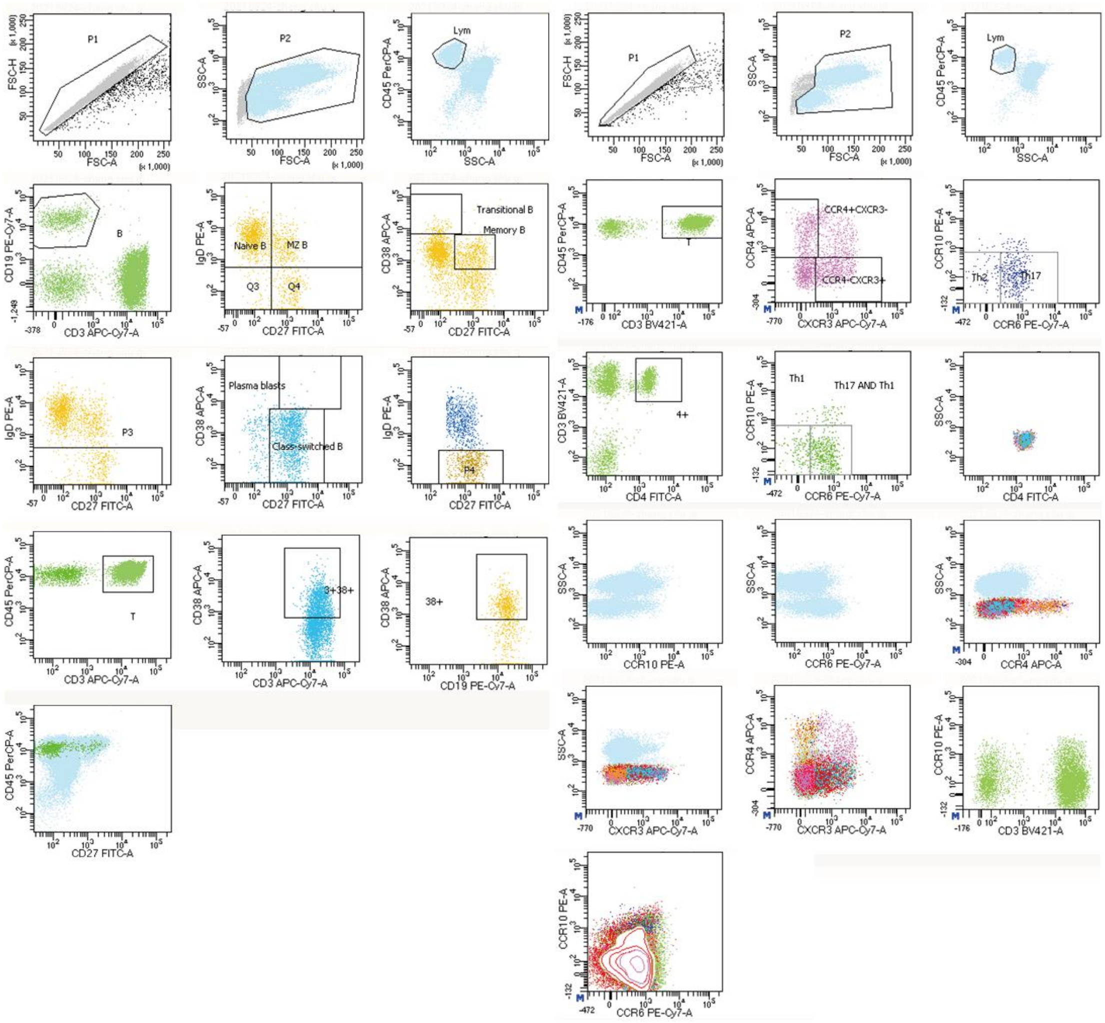

Supplementary Figure 29 | Flow cytometry analysis of B cell and Th cell subsets in patient sample H4.

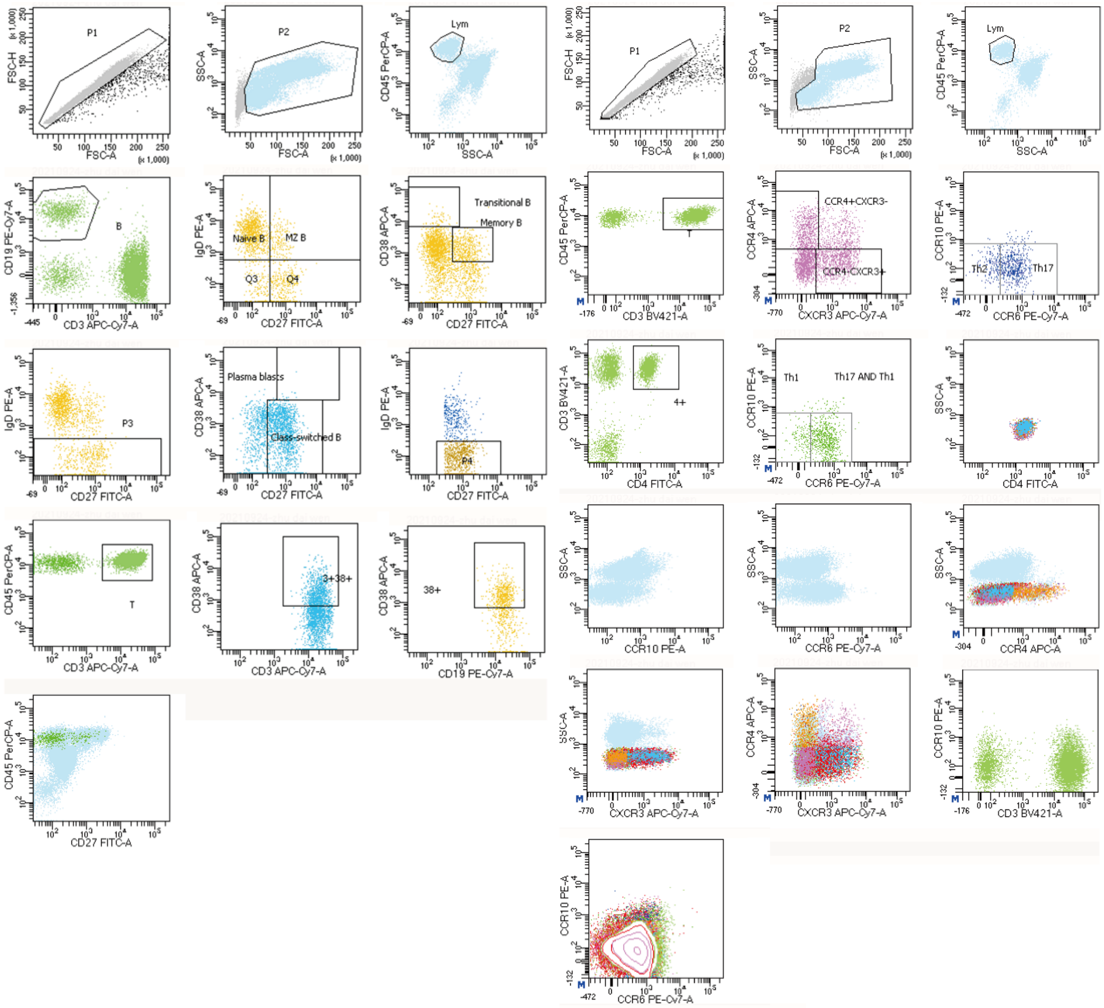

Supplementary Figure 30 | Flow cytometry analysis of B cell and Th cell subsets in patient sample H5.

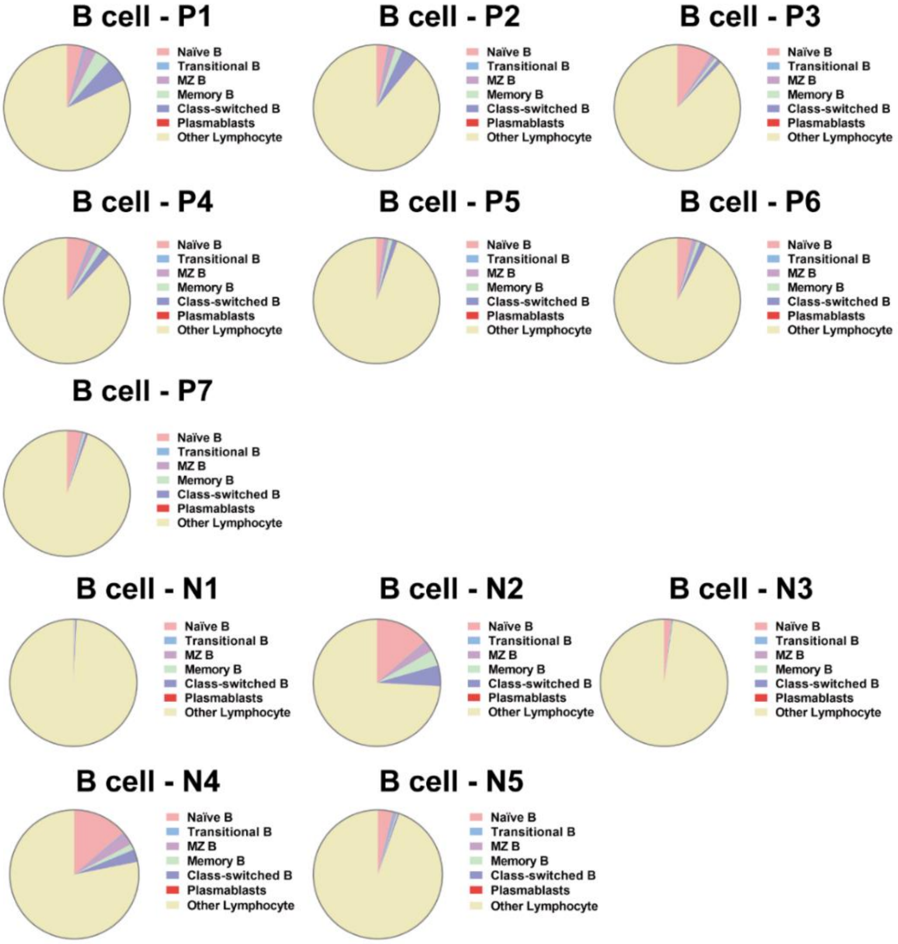

Supplementary Figure 31 | The B cell subsets distribution in lymphocytes of seven IgG$^+$/IgM$^+$ KTRs (P1-P7) and five IgG$^-$/IgM$^-$ KTRs (N1-N5).

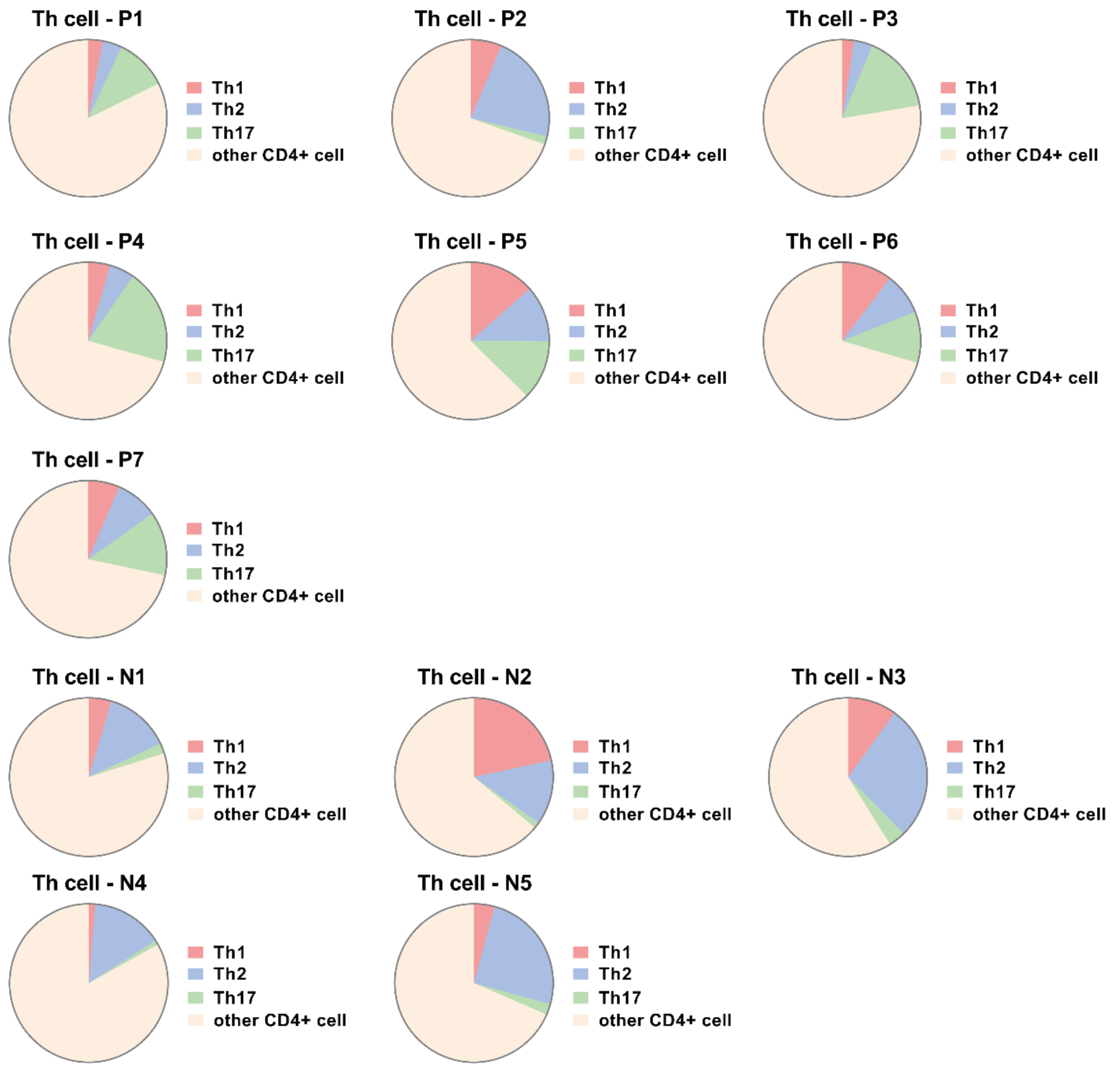

Supplementary Figure 32 | The Th cell subsets distribution in $CD4^+$ cells of seven IgG^+/IgM^+ KTRs (P1-P7) and five IgG^-/IgM^- KTRs (N1-N5).

Supplementary Table 3. The description and phenotypes of investigated B cell subtypes and the medians with minimum and maximum of proportional in two KTR cohorts.

Description	Phenotype	Denominator	Proportional (%) Median (Range)	
			Cohort 1 (IgG^+/IgM^+)	Cohort 2 (IgG^-/IgM^-)
Lymphocyte	$CD45^+$ SS (low) FS (low)	White Blood Cells	16.6 (11.6-55.6)	79.8 (72.9-94.4)
T cells	$CD3^+$	Lymphocytes	68 (50.2-86.2)	75.7 (42.1-84.2)
B cells	$CD19^+$ $CD3^-$	Lymphocytes	11 (4.9-14.3)	5 (0.9-22.4)
Naive B cells	$CD19^+$ $CD27^-$ IgD^+	B cells	54.6 (26.0-78.7)	63.6 (21.0-78.4)
Transitional B cells	$CD19^+$ $CD27^-$ $CD38^{high}$ IgM^+ $CD24^+$	B cells	2.9 (1.7-4.1)	1.4 (0.8-11.2)
MZ B cells	$CD19^+$ $CD27^+$ IgD^+	B cells	13.5 (5.5-23.8)	7.3 (1.2-14.1)
Memory B cells	$CD19^+$ $CD27^+$ $CD38^{dim}$	B cells	13.7 (6.2-27.7)	7.1 (5.0-32.1)
Class-switched B cells	$CD19^+$ $CD27^+$ $CD38^{dim}$ IgD^- IgM^-	B cells	23.6 (9.1-43.3)	14.5 (7.8-32.1)
Plasmablasts	$CD19^+$ $CD27^{high}$ $CD38^{high}$ IgD^- IgM^-	B cells	0.5 (0.2-1.1)	0.1 (0.0-1.2)
Th1	$CD3^+$ $CD4^+$ $CCR4^-$ $CXCR3^+$ $CCR6^+$	$CD4^+$ cells	6.4 (2.5-13.5)	4.6 (1.3-21.6)
Th2	$CD3^+$ $CD4^+$ $CCR4^+$ $CXCR3^-$ $CCR6^-$	$CD4^+$ cells	8.6 (3.8-22.3)	14.7 (13.2-27.6)
Th17	$CD3^+$ $CD4^+$ $CCR4^+$ $CXCR3^-$ $CCR6^+$ $CCR10^-$	$CD4^+$ cells	12.2 (1.7-19.6)	2.1 (0.9-3.5)

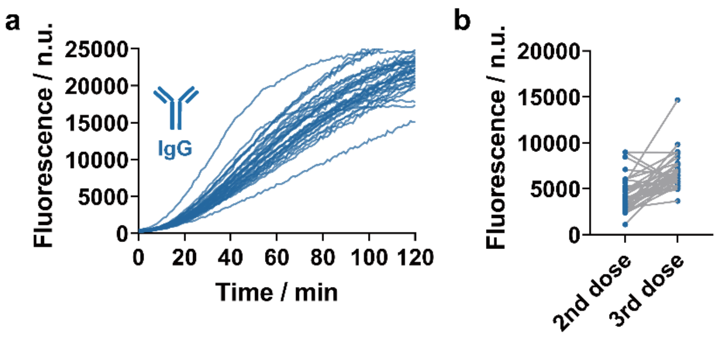

Supplementary Figure 33 | UCAD analysis of KTRs with the 3rd dose of vaccine. (**a**) Kinetic curves for the detection of anti-RBD IgG in the 33 KTRs who received the 3rd dose of inactivated COVID-19 vaccine. (**b**) Comparison of anti-RBD IgG signals of the 33 KTRs received the 2nd and 3rd dose of vaccine.
