# Supplementary material for: A CRISPR-based ultrasensitive assay detects attomolar concentrations of SARS-CoV-2 antibodies in clinical samples
Source: Nat Commun. 2022 Aug 9;13:4667. doi: 10.1038/s41467-022-32371-4 (PMC9361972; doi:10.1038/s41467-022-32371-4)
Supplement: Supplementary file 3 — Description of additional Supplementary File [file 41467_2022_32371_MOESM3_ESM.pdf]

Description of Additional Supplementary Data Files

Supplementary Data Set 1. Supplementary excel file lists clinical test results of 65 vaccinated healthy participates.

Supplementary Data Set 2. Supplementary excel file lists clinical test results of 55 unvaccinated participants.

Supplementary Data Set 3. Supplementary excel file lists vaccination information of 85 KTRs.
