# Supplementary material for: A CRISPR-based ultrasensitive assay detects attomolar concentrations of SARS-CoV-2 antibodies in clinical samples
Source: Nat Commun. 2022 Aug 9;13:4667. doi: 10.1038/s41467-022-32371-4 (PMC9361972; doi:10.1038/s41467-022-32371-4)
Supplement: Supplementary file 7 — Reporting Summary [file 41467_2022_32371_MOESM7_ESM.pdf]

nature portfolio

Corresponding author(s): Feng Li, Tao Lin

Last updated by author(s): Jul 14, 2022

Reporting Summary

Nature Portfolio wishes to improve the reproducibility of the work that we publish. This form provides structure for consistency and transparency in reporting. For further information on Nature Portfolio policies, see our Editorial Policies and the Editorial Policy Checklist.

Statistics

For all statistical analyses, confirm that the following items are present in the figure legend, table legend, main text, or Methods section.

n/a	Confirmed	
☐	☒	The exact sample size (n) for each experimental group/condition, given as a discrete number and unit of measurement
☐	☒	A statement on whether measurements were taken from distinct samples or whether the same sample was measured repeatedly
☐	☒	The statistical test(s) used AND whether they are one- or two-sided<br>*Only common tests should be described solely by name; describe more complex techniques in the Methods section.*
☒	☐	A description of all covariates tested
☒	☐	A description of any assumptions or corrections, such as tests of normality and adjustment for multiple comparisons
☐	☒	A full description of the statistical parameters including central tendency (e.g. means) or other basic estimates (e.g. regression coefficient) AND variation (e.g. standard deviation) or associated estimates of uncertainty (e.g. confidence intervals)
☐	☒	For null hypothesis testing, the test statistic (e.g. F, t, r) with confidence intervals, effect sizes, degrees of freedom and P value noted<br>*Give P values as exact values whenever suitable.*
☒	☐	For Bayesian analysis, information on the choice of priors and Markov chain Monte Carlo settings
☒	☐	For hierarchical and complex designs, identification of the appropriate level for tests and full reporting of outcomes
☒	☐	Estimates of effect sizes (e.g. Cohen's d, Pearson's r), indicating how they were calculated

Our web collection on statistics for biologists contains articles on many of the points above.

Software and code

Policy information about availability of computer code

Data collection	The raw fluorescence data were collected by Cytation 5 multimode microplate reader. Cell characterization was performed using BD FACSCanto™ II Cytometer.
Data analysis	OligoAnalyzer (IDT) is an web-based DNA calculator provided by Integrated DNA Technologies, Inc., and can be freely access in https://sg.idtdna.com/calc/analyzer. The raw fluorescence data were analyzed by Microsoft 365 Excel and GraphPad 8.0.1. Thermodynamic data and melting temperatures were estimated using OligoAnalyzer (IDT). Flow cytometry data were analyzed by BD FACSDiva™ v9.0.

For manuscripts utilizing custom algorithms or software that are central to the research but not yet described in published literature, software must be made available to editors and reviewers. We strongly encourage code deposition in a community repository (e.g. GitHub). See the Nature Portfolio guidelines for submitting code & software for further information.

Data

Policy information about availability of data

All manuscripts must include a data availability statement. This statement should provide the following information, where applicable:

- Accession codes, unique identifiers, or web links for publicly available datasets
- A description of any restrictions on data availability
- For clinical datasets or third party data, please ensure that the statement adheres to our policy

All data is available in the main text or the supplementary information text and files. The raw fluorescence data for all UCAD tests, unprocessed gel image, and data for flow cytometric analyses are provided in the Source data file.

Field-specific reporting

Please select the one below that is the best fit for your research. If you are not sure, read the appropriate sections before making your selection.

☒ Life sciences ☐ Behavioural & social sciences ☐ Ecological, evolutionary & environmental sciences

For a reference copy of the document with all sections, see nature.com/documents/nr-reporting-summary-flat.pdf

Life sciences study design

All studies must disclose on these points even when the disclosure is negative.

Sample size	No sample size calculation was performed. The sample sizes of both healthy participants (n = 137) and KTRs (n = 85) were determined by the sample availability and were sufficient to draw statistically meaningful conclusions.
Data exclusions	No data exclusion for clinical samples.
Replication	Technical replications (n = 2) were successfully performed for in vitro tests in Figures 1, 2, 3, S1-S4, S6-S9 as shown in Source Data file. No replication was performed for clinical sera samples, because of the limited sample volumes for UCAD analyses.
Randomization	All samples were tested with no random selection in order to evaluate the detection performance of our estimated method.
Blinding	Clinical samples were tested by an individual who was blinded to the CLIA results.

Reporting for specific materials, systems and methods

We require information from authors about some types of materials, experimental systems and methods used in many studies. Here, indicate whether each material, system or method listed is relevant to your study. If you are not sure if a list item applies to your research, read the appropriate section before selecting a response.

Materials & experimental systems

n/a	Involved in the study	
☐	☒	Antibodies
☒	☐	Eukaryotic cell lines
☒	☐	Palaeontology and archaeology
☒	☐	Animals and other organisms
☐	☒	Human research participants
☒	☐	Clinical data
☒	☐	Dual use research of concern

Methods

n/a	Involved in the study	
☒	☐	ChIP-seq
☐	☒	Flow cytometry
☒	☐	MRI-based neuroimaging

Antibodies

Antibodies used	SARS-CoV-2 Spike RBD human monoclonal antibody: TA190325, clone name: OTIH401, Origene, Beijing, China.<br>SARS-CoV-2 N protein human monoclonal antibody: TA190323, clone name: OTIH1G5, Origene, Beijing, China.<br>SARS-CoV-2 Spike RBD rabbit polyclonal antibody: A20135, ABclonal Biotech, Wuhan, China.<br>Mers-CoV Spike RBD human monoclonal antibody: Q3731380, clone name: M336, EMD Millipore, Darmstadt, Germany<br>SARS-CoV-2 Spike RBD human monoclonal antibody (Delta specific): SPD-M370, clone name: AM110, ACRObiosystems, Beijing, China<br>SARS-CoV-2 Spike RBD human monoclonal antibody (Omicron specific): SPD-M415, clone name: AS113, ACRObiosystems, Beijing, China<br>Digoxin rabbit monoclonal antibody: A20267, ABclonal Biotech, Wuhan, China.<br>Biotin-conjugated goat anti-human IgG: D110152-0100, Sangon Biotech., Shanghai, China.<br>Biotin-conjugated goat anti-human IgM: D110159-0100, Sangon Biotech., Shanghai, China.<br>Anti-FITC rabbit polyclonal antibody ： D110003-0200, Sangon Biotech., Shanghai, China.
Validation	All antibodies were validated as described on the websites of their suppliers:<br>SARS-CoV-2 Spike RBD human monoclonal antibody: TA190325<br>https://www.origene.com/catalog/antibodies/primary-antibodies/ta190325/sars-cov-2-spike-protein-rbd-monoclonal-antibody<br><br>SARS-CoV-2 N protein human monoclonal antibody: TA190323<br>https://www.origene.com/catalog/antibodies/primary-antibodies/ta190323/sars-cov-2-n-protein-human-monoclonal-antibody<br><br>SARS-CoV-2 Spike RBD rabbit polyclonal antibody: A20135<br>https://abclonal.com.cn/catalog/A20135

Mers-CoV Spike RBD human monoclonal antibody: Q3731380
https://www.merckmillipore.com/LU/fr/product/Anti-MERS-CoV-RBD-Antibody-clone-m336,MM_NF-MABF3060-25UG

SARS-CoV-2 Spike RBD human monoclonal antibody (Delta specific) SPD-M370
https://www.acrobiosystems.com/P4590-Anti-SARS-CoV-2-Spike-RBD-Antibody-Chimeric-mAb-Human-IgG1-%28AM110%29-%28Delta-Specific%29.html

SARS-CoV-2 Spike RBD human monoclonal antibody (Omicron specific) SPD-M415
https://www.acrobiosystems.com/P4931-Anti-SARS-CoV-2-Spike-RBD-Antibody-Chimeric-mAb-Human-IgG1-%28AS113%29-%28Omicron-Specific%29.html

Digoxin rabbit monoclonal antibody: A20267
https://abclonal.com.cn/catalog/A20267

Biotin-conjugated goat anti-human IgG: D110152-0100
https://www.sangon.com/productDetail?productInfo.code=D110152

Biotin-conjugated goat anti-human IgG: D110159-0100
https://www.sangon.com/productDetail?productInfo.code=D110159

Anti-FITC rabbit polyclonal antibody: D110003-0200
https://www.sangon.com/productDetail?productInfo.code=D110003

Human research participants

Policy information about studies involving human research participants

Population characteristics: Adult kidney transplant recipients from West China Hospital, Sichuan University with stable renal function, without any episode of rejection or infection in last 3 months were enrolled. Population characteristics, including gender, age, past record of COVID-19 vaccination and seroconversion test were listed in Supplementary Data.

Recruitment: Serum samples were collected based on time, diagnosis and participant consents. All participates involved voluntarily and there is no participant compensation. No further selection was made by the researchers. After clear statement of the goal of the present study and how it is manipulated, we got signed consents from all participants.

Ethics oversight: This study was approved by the Ethics Committee of West China Hospital, Sichuan University (NO: 2021-110)

Note that full information on the approval of the study protocol must also be provided in the manuscript.

Flow Cytometry

Plots

Confirm that:

- [x] The axis labels state the marker and fluorochrome used (e.g. CD4-FITC).
- [x] The axis scales are clearly visible. Include numbers along axes only for bottom left plot of group (a 'group' is an analysis of identical markers).
- [x] All plots are contour plots with outliers or pseudocolor plots.
- [x] A numerical value for number of cells or percentage (with statistics) is provided.

Methodology

Sample preparation: A 2 ml EDTAanticoagulated blood sample was collected by venipuncture for antibody staining. Whole blood was used and staining for lymphocyte surface markers was performed after red cell lysis, according to a standard flow cytometric multicolor protocol.

Instrument: CantoII flow cytometer (BD, 3-laser configuration)

Software: BD FACSDiva™

Cell population abundance: The blood cell analyzer was used to count the peripheral blood samples of the patients, and the amount of samples was adjusted according to the cell count, and then the corresponding amount of cells was added according to the detection items. About 1X106~107 cells were added to each tube. The number of cells to be obtained requires that the total number of lymphocytes should not be less than 200,000, and the rare target cell population should not be less than 100 cells in the positive gate.For cryopreserved samples, activity testing is required after cell resuscitation. Samples with cell viability below 75% are not subject to flow cytometry.

Gating strategy: The gating strategy is FSC/SSC, 45/SSC, CD45/CD19, CD45/CD3, CD3/CD19, CD3/CD16+56. Using homotypic control or cells of similar size as negative control, there is a clear boundary between strongly expressed and moderately expressed antigens and

negative cells, with little overlap with each other. For weakly expressed and heterogeneously expressed antigens, negative control cells are strictly applied, and MFI values are used as reference when necessary.

☒ Tick this box to confirm that a figure exemplifying the gating strategy is provided in the Supplementary Information.
